# Variations in the uptake of telemental health technologies in community and crisis mental health services during the early pandemic: a survey of mental health professionals in the UK

**DOI:** 10.1186/s12888-022-04385-1

**Published:** 2022-12-09

**Authors:** Luke Sheridan Rains, Christian Dalton-Locke, Sabine Landau, Justin J. Needle, Sonia Johnson

**Affiliations:** 1 Division of Psychiatry, NIHR Mental Health Policy Research Unit, University College London, London, UK; 2 Department of Biostatistics and Health Informatics, NIHR Mental Health Policy Research Unit, Institute of Psychiatry, Psychology & Neuroscience, King’s College London, London, UK; 3 Centre for Health Services Research, School of Health Sciences, City, University of London, London, UK; 4 Camden and Islington NHS Foundation Trust, London, UK

**Keywords:** Telemental health, Videoconferencing, Telehealth, Covid-19, Pandemic, Lockdown, Community Mental Health Services

## Abstract

**Background:**

One of the many challenges faced by mental health services during the COVID-19 pandemic was how to deliver care during lockdown. In community and crisis services, this often meant rapidly adopting or expanding the use of telemental health technologies, including phone and video calls. The aim of this study is to explore variations in use and report staff views of such technologies during the early stages of the pandemic. The primary analysis compared rates of use between professions, demographic groups, genders, regions, and crisis and community services.

**Methods:**

We used data from an online survey conducted by the Mental Health Policy Research Unit in Spring 2020 regarding the impact of the pandemic on mental healthcare in the United Kingdom. We included quantitative data from all professional groups working in community or crisis services providing care to working age adults, including general and specialist services. Our outcome of interest was the percentage of clients whom clinicians primarily interacted with via videocall. We also collected demographics and professional characteristics such as the type of mental health service respondents worked in. In addition, we explored respondents’ views and experiences of telemental health as a medium for providing care.

**Results:**

978 participants were included in the primary analysis (834 provided outcome data for community services, 193 for crisis services). In community services, virtually all staff reported stopping some or all face-to-face appointments following the onset of the pandemic, with a large majority using video or phone call appointments where possible instead. Telemental health use was higher in community than in crisis services, and amongst professionals who mainly provided psychotherapy or peer support than in other groups. There was also evidence of use being lower in regions in Northern England, Scotland, and Wales than elsewhere. There was no evidence of an association with staff gender, age, or ethnicity. Staff were generally positive about telemental health and intended to make more use of technologies following the pandemic. However, significant barriers to its use were also reported, often involving skills and available infrastructure.

**Conclusions:**

Despite its rapid implementation, telemental health was viewed positively by clinicians who saw it as an effective alternative to face-to-face appointments in some contexts, including during the pandemic. However, adoption of the technology also has the potential to exacerbate existing or create new inequalities without effective management of training and infrastructure needs.

**Supplementary Information:**

The online version contains supplementary material available at 10.1186/s12888-022-04385-1.

## Background

Following the onset of the COVID-19 pandemic in the early months of 2020, most countries have experienced a severe disruption of mental health service delivery [1] at a time of increased demand due to the adverse mental health consequences of the pandemic [2, 3].

Mental healthcare providers responded to the challenges caused by social distancing requirements, ‘stay at home’ orders and national lockdowns in many ways, including the rapid and widely documented shift to remote delivery of mental health services - not commonly employed before the pandemic - to replace in-person consultations [1, 4, 5].

### Telemental health

Telemental health, defined as “the provision of behavioural and/or mental healthcare services using technological modalities in lieu of, or in addition to, traditional face-to-face methods” [6], including video conferencing, telephone, email, or text messaging, has been central to continuing assessment and support in hospital, community and other settings [5, 7]. Numerous research studies conducted both before and during the pandemic have reported evidence of the usefulness of telemental health in reducing treatment gaps and improving access to care for a range of service users [8–10]. Findings suggest that, across the full range of settings and patient populations, synchronous modalities such as telephone and, especially, videoconferencing can be comparable under the right conditions to face-to-face delivery in terms of quality of care, therapeutic relationships, reliability of clinical assessments, symptom severity, treatment outcomes, access, attendance and adherence [11–16]. A number of studies also suggest that telemental health may be more cost-effective than face-to-face care, although the economic evidence remains limited [11, 14].

High levels of service user acceptance and satisfaction with telemental health services, again comparable with face-to-face care, have been reported across a range of populations [10, 11, 17]. Overall, many service users, especially in contexts where they have actively chosen a telemental health programme, value the convenience of being able to access services remotely, and this had made care more accessible to those who previously found it difficult to engage with face-to-face support, for example, due to travel-related costs or family or work commitments. However, service users have also identified a number of challenges in relation to remote delivery, such as access to technology and a stable internet connection, ensuring privacy and confidentiality for those who lack private space or find participating in discussions from home intrusive, and maintaining concentration during remote sessions. These challenges have been especially prominent in relation to the widespread rapid implementation of telemental health as an emergency response to the COVID-19 pandemic [5, 12, 18, 19].

### Variations in the use of telemental health during the pandemic

Despite high levels of adoption of telemental health during the pandemic, its utilisation has varied substantially both between and within countries [1, 7]. Significant variations exist in the feasibility and appropriateness of remote care delivery across different groups of service users, potentially exacerbating inequalities in access to services. For example, telemental health is not suitable for delivering types of therapy, such as exposure therapy and role play, which require a physical presence [20], and may be less appropriate for certain groups, such as younger children and those with trauma, severe anxiety, learning difficulties, autism, or cognitive impairment [21].

Specific concerns relating to the use of telemental health have also been reported for a number of populations, such as people whose conditions are exacerbated by pandemic-related anxieties and social disruption; those experiencing loneliness, domestic abuse, or family conflict; those who experience difficulties engaging with remote care; and those who are digitally excluded due to significant social disadvantage or limited technological access and expertise [7, 12, 18]. Issues relating to access to technology have been reported particularly for some older adults [22] and service users from lower socio-economic backgrounds [23], or with diagnoses such as schizophrenia [24]. Although other studies have found good acceptability amongst some older adults [25]. These findings are consistent with research conducted prior to the pandemic [26, 27].

Other types of patterns in the use of telemental health have also been observed during the pandemic. For example, variations have been reported across care settings, clinical populations served, urban and rural areas, and mental health professionals; depending on their level of training, quality of organisational support and leadership, therapeutic orientation and individual and practice characteristics, including the ability and willingness to prepare and support service users in using remote technology [12, 28, 29]. However, research on variations in telemental health use during the pandemic within the United Kingdom remains limited.

### Clinician attitudes towards telemental health

Prior to the COVID-19 pandemic, clinician experiences of and attitudes towards synchronous telemental health also appear to have been largely positive, with professionals finding it an effective, flexible, and acceptable means of delivering care, and recognise its potential to enhance communication within and between mental health teams [11]. However, reactions to this technology during the pandemic have been more equivocal, and there have been reports of significant challenges arising from its widespread and rapid adoption in place of most or all face-to-face communication [5, 11, 30, 31]. Despite these challenges and reports from clinicians that face-to-face contacts are ideal for both assessment and treatment, many would nonetheless be willing to continue with some aspects of telemental health care delivery after the pandemic period: further research into its longer-term implementation, sustainability, and acceptability is needed [12, 17, 32].

A number of facilitators for clinician uptake of telemental health have been identified, including previous experience of using online (especially video-based) platforms for delivering care and confidence in using them, being an experienced clinician, the availability of adequate support and supervision, effective leadership, clear communication, optimising physical space for comfort and privacy, and ensuring time away from the computer [5, 12]. However, a number of barriers to the delivery of high-quality remote care have been widely identified. Mental health staff have reported concerns relating to limited technological infrastructure within services, a lack of clear protocols, inadequate support and training, adherence to privacy regulations and the management of risk and safeguarding of service users while using remote methods of care [5, 12, 28, 33].

In terms of quality of therapeutic relationships, while the transition to telemental health has brought benefits, such as helping clinicians engage better with some service users who find face-to-face meetings difficult [12], a number of challenges to building and maintaining good therapeutic relationships remotely have been identified. These include difficulties in assessing service users’ mental health symptoms, emotions, and physical indicators of mental health status (such as hygiene and physical symptoms of opioid withdrawal), reduced feelings of connection to and empathy with service users, challenges in maintaining service user engagement, and possible misunderstandings due to, for example, the inability to pick up on nonverbal signals and reactions [12, 34, 35].

Despite these issues, telemental health has proved a valuable tool in helping mental health services respond to the COVID-19 emergency. However, the study authors are not aware of any other published studies examining telemental health adoption in the UK during the early pandemic. Variable adoption between settings and clinicians, and the impediments reported, need to be better understood in order to inform preparedness for any future emergency, and to identify settings and contexts in which telemental health may continue to be useful beyond the pandemic. Staff attitudes and the ways in which these vary are an important component in developing such an understanding.

### Objectives

In early 2020, we (the NIHR Mental Health Policy Research Unit (MHPRU)) conducted a survey of mental health professionals across the United Kingdom in order to assess the impact of the pandemic on mental health services [7], including their reports on the rapid adoption of telemental health that had occurred during the early stages of the pandemic. Using quantitative data from this survey, the aims of the present study are: (1) to describe the utilization rates of telemental health modalities during the early stages of “lockdown” in response to the Covid-19 pandemic, (2) describe the experiences and opinions of professionals regarding this modality, and (3) investigate the factors that influence the use of this modality.

## Methods

Our study is a sub-group analysis of data from a cross-sectional, mixed methods survey of UK mental health care staff across all regions and sectors, conducted between 22nd April and 12th May 2020. Approval was received from King’s College London research ethics committee study (MRA-19/20–18,372). Both the original and present studies were conducted in accordance with relevant guidelines and regulations. The survey was disseminated via social media (mainly Twitter), professional networks (for example, the Mental Health Nurse Academics UK, Unite the Union, Royal College of Psychiatrists, and Royal College of Nurses), and relevant mental health-focused bodies (for example, the Centre for Mental Health and the Association of Mental Health Providers). Main descriptive findings from the survey were reported by Johnson et al. [7]. In this study we focus on the sample included in the sub-group analyses on which we report.

### Participants

The sample for the present study comprised survey respondents who worked in: 1) community teams and psychological treatment services, including secondary mental health teams providing all types of continuing care (community mental health teams, rehabilitation teams, assertive outreach teams and Early Intervention in Psychosis (EIS) services, and services delivering psychological treatment, such as Improving Access to Psychological Therapies (IAPT) teams); 2) crisis assessment services, including crisis resolution and home treatment teams, walk-in crisis assessment centres and psychiatric liaison services based in emergency departments. We included data from all professional groups working in these settings, including clinical or counselling psychologists, nurses, occupational therapists, other qualified therapists, peer support workers, psychiatrists, social workers, and managers in mental health services. We excluded data from specialist services for older adults or children and adolescents as we anticipated respondents using these services would have different experiences of telemental health.

### Questionnaire content

The questionnaire was developed with input from a group of around 40 people, including clinicians, researchers and people with lived experience. It contained a mixture of multiple-choice and free-text questions and was divided into three main sections: 1) current work challenges; 2) service users’ and carers’ problems; and 3) sources of help. The survey contained some sections presented to all participants and others that were only for staff working in specific settings. Settings included continuing community care and crisis care services, as well as services for older adults, children and young people, perinatal, forensic, intellectual disabilities, drug and alcohol problems, eating disorders, and day services. The survey contained 99 questions that were presented to all participants, and between 3 and 64 questions for each setting. Depending on how many settings they worked in, participants could be asked between 99 and 277 questions in total. Completing the survey was estimated to take between 15 and 30 minutes depending on the detail provided to free-text questions. A copy of the survey is available in the supplementary materials, which shows the filtering rules for the questions.

Participants working in community and crisis services were asked whether they were using telephone calls or video consultations (including WhatsApp video, Zoom, Microsoft Teams or any other video call platform) to replace some or all face-to-face meetings with service users, to which the participant could answer by selecting *Yes* or *No*. Participants who selected *Yes* were then asked a series of questions relating to telemental health. The primary outcome for the current study was the estimated percentage of service users that participants see with whom they now mainly have contact by video call, which was used as an indicator of telemental health technology adoption.

The questionnaire also included 21 statements about staff use of telemental health and their views and experiences of it as a medium for providing care. For 17 of the statements, participants used a five-point Likert scale to indicate their level of agreement (*Strongly disagree, Disagree, Neither agree nor disagree, Agree, Strongly agree*); while for the other four items, participants again used a five-point Likert scale to rate the degree of relevance (*Not relevant, Slightly, Moderately, Very, Extremely relevant*). Staff working in community or psychological treatment services were also asked to rate their level of agreement or disagreement with an additional two statements relating specifically to delivering psychological treatment using telemental health. In addition, participants were asked questions regarding their demographic and other characteristics: gender, age, ethnicity, profession, type of mental health service, and geographical location.

### Data analysis

Descriptive statistics were generated to summarise participant demographics and other characteristics, such as their current profession, speciality, work setting, and region for the whole sample as well as the sub-sample of those included in the primary analysis. These were also generated for survey items capturing staff views and experiences of telemental health technologies, including telephone and video call technology used for communication with service users or between staff, using whole sample data. Our outcome of interest was the clinician estimates of the percentage of their service users that they mainly contact via video call, divided into four levels: none (0%), low (1–20%), medium (21–60%), and high (61–100%). Items related to remote working, as well as demographics, type of service, profession, and regions, were used to explore patterns of use.

We used ordinal logistic regression mixed-effects modelling to test for variables associated with this outcome: extent of use of video calls for service user contacts. The following variables were considered as potential predictor variables: age, gender, ethnicity, type of service, profession, and region. We used mixed-effects modelling as some outcome data came from participants working in both crisis and community settings, potentially leading to correlations between multiple outcome values reported by the same participant but for different settings. The mixed-effects modelling allowed us to account for this by modelling a random intercept at the respondent level. We initially fitted a separate model for each predictor variable. For each categorical predictor, the reference category was the one associated with the highest median percentage of service users contacted via telemental health modalities. Subsequently, we fitted a single model with all independent variables that met a threshold of *p* < 0.1 during the initial analyses. We used backward model selection at the *p* = 0.05 level to identify key variables for inclusion within a final model. Wald tests were used to test the significance of individual predictor variables in the final model. Interaction effects between profession-related predictor variables (region, profession, and work setting) and demographics (age, gender, ethnicity) were further explored based on this model, and were found to be non-significant at the *p* = 0.05 level. All analyses were performed using STATA v.16 [36].

## Results

In total, 3712 people completed the original survey [7]. Of these, 40.5% (1504/3712) reported working in community services only, 7.0% (261/3712) in crisis services only, and 4.7% (174/3712) reported working in both, giving a total sample of 1939 participants (52.2% of all survey respondents). 30.0% (1114/3712) of survey respondents reported working in neither and 17.8% (659/3712) did not complete this item on the survey, so were not included in the present study.

### Participant demographics and professional characteristics

Table 1 presents participant demographic and other participant characteristics for both the whole sample, and the sample included in the primary analyses. Most characteristics were similar between these samples. Between 55.8% (1081/1939) and 58.4% (1133/1939) of the whole sample provided demographic data, of which 81.7% were female (926/1133), 80.1% were aged 25–54 (900/1123), and 87.7% gave their ethnicity as white (948/1081). A higher proportion provided data for other characteristics, with between 91.7% (1780/1939) to 95.6% (1853/1939) responding. Of these, 32.4% (576/1780) of participants reported being based in London, 14.5% (258/1780) in Northwest England, 12.5% (223/1780) in Southeast England, with 26.9% (478/1780) in other regions across England, and 9.6% (171/1780) in Scotland and 4.2% (74/1780) in Wales. 86.5% (1678/1939) reported working in community services, while 22.4% (435/1939) reported working in crisis services. In the whole sample, 28.2% (524/1853) were nurses, 17.5% (338/1853) were clinical or counselling psychologists, 17.7% (328/1853) were occupational or other qualified therapists, 12.1% (234/1853) were psychiatrists, and 14.3% (275/1853) worked in non-qualified professions, management, or selected ‘other’, and were not included in the primary analyses.

**Table 1:** Demographics and service details for participants working in community and crisis services

Variable	Value	Sample with primary outcome data n/N (%)	Whole sample n/N (%)
Gender	Male	167/923 (18.1%)	207/1133 (18.3%)
	Female	756/923 (81.9%)	926/1133 (81.7%)
Age	Under 25	24/918 (2.6%)	29/1123 (2.6%)
	25–34	205/918 (22.3%)	251/1123 (22.4%)
	35–44	237/918 (25.8%)	288/1123 (25.7%)
	45–54	302/918 (32.9%)	361/1123 (32.2%)
	55 or over	150/918 (16.3%)	194/1123 (17.3%)
Ethnicity	White	790/891 (88.7%)	948/1081 (87.7%)
	Asian	51/891 (5.7%)	64/1081 (5.9%)
	Black	15/891 (1.7%)	24/1081 (2.2%)
	Mixed/Other	35/891 (3.9%)	45/1081 (4.2%)
Profession	Clinical or counselling Psychologist	322/881 (36.6%)	338/1853 (17.5%)
	Nurse	276/881 (31.3%)	524/1853 (28.2%)
	Occupational or Other qualified Therapist	73/881 (8.3%)	328/1853 (17.0%)
	Peer Support Worker	20/881 (2.3%)	51/1853 (2.7%)
	Psychiatrist	131/881 (14.5%)	234/1853 (12.1%)
	Social Worker	59/881 (6.7%)	103/1853 (5.3%)
	Other (non-clinical)	0/881 (0.0%)	275/1853 (14.3%)
Type of Service	Community services	834/978 (85.3%)	1678/1939 (86.5%)
	Crisis services	193/978 (19.7%)	435/1939 (22.4%)
Region	West Midlands	58/966 (6.0%)	93/1780 (5.2%)
	East Midlands	39/966 (4.0%)	59/1780 (3.3%)
	East of England	63/966 (6.5%)	113/1780 (6.4%)
	London	298/966 (30.9%)	576/1780 (32.4%)
	Northeast	34/966 (3.5%)	60/1780 (3.4%)
	Northwest	142/966 (14.7%)	258/1780 (14.5%)
	Southeast	119/966 (12.3%)	223/1780 (12.5%)
	Southwest	50/966 (5.2%)	91/1780 (5.1%)
	Yorkshire and The Humber	27/966 (5.2%)	62/1780 (3.5%)
	Scotland	101/966 (10.5%)	171/1780 (9.6%)
	Wales	35/966 (3.6%)	74/1780 (4.2%)

### Variations in use of telemental health technologies between demographic and professional groups

**Fig. 1:**
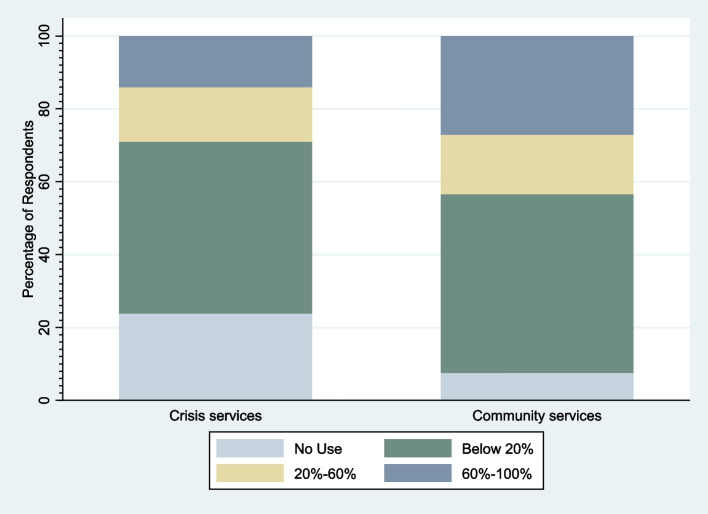
Percentage of service users participants mainly support using video call by clinical setting

**Fig. 2:**
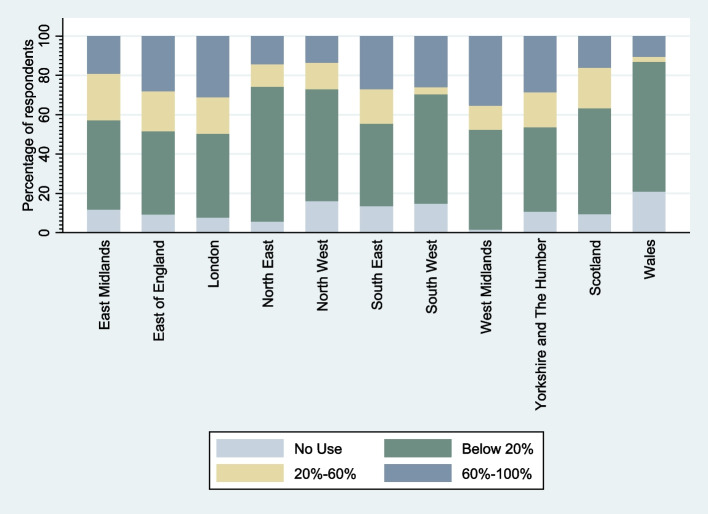
Percentage of service users participants mainly support using video call by region

**Fig. 3:**
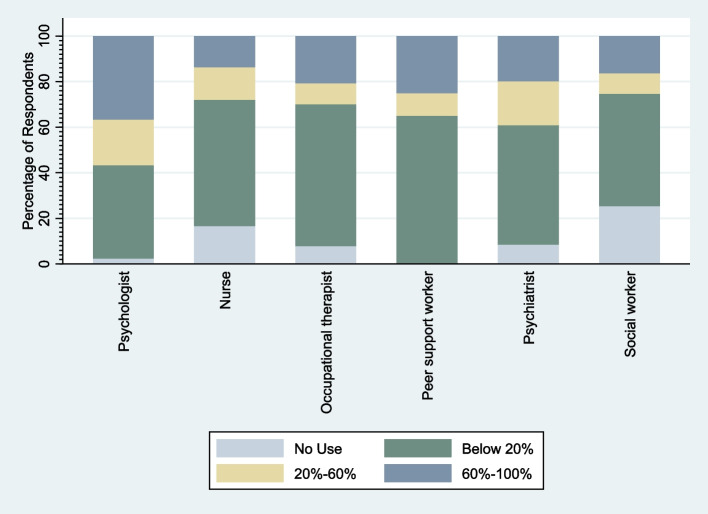
Percentage of service users participants mainly support using video call by profession

Out of the whole sample of 1939 participants, 978 provided primary outcome data (proportion of service users they mainly support using video calls). The remaining participants did not complete this item in the questionnaire. Of those who provided primary outcome data, 193 worked in crisis services and 834 worked in community services. Results of univariate analyses (Table 2) indicate that level of use of telemental health technology varied by region (*p* = 0.02), type of service (*p* = < 0.01), and profession (*p* = < 0.01), but there was no evidence of variation in use across demographic (groups). Overall, use was higher in community compared to crisis services (Fig. 1). Effect of region was also significant overall, but evidence of variation varied between regions. As shown in Fig. 2, use was most extensive in the West Midlands (reference group) and was relatively high in the East Midlands and the Southeast of England, including London, but was comparatively low in the Northeast and Northwest of England, as well as Scotland and Wales. Clinical and counselling psychologists used telemental health technologies the most (Fig. 3), while nurses, occupational and other therapists, psychiatrists, and social workers used it much less.

**Table 2:** Results of univariate analyses

Variable (reference category) (*N=*)	Value (n)	Odds Ratio (95% CI)	*P* value of overall test
Gender (*N =* 923)	Male (*n =* 167)	Reference	0.44
	Female (*n =* 756)	1.45 (0.57; 3.71)	
Age (*N =* 918)	18–24 (*n =* 24)	Reference	0.98
	25–34 (*n =* 205)	0.97 (0.08; 12.31)	
	35–44 (*n =* 237)	1.10 (0.09; 13.82)	
	45–54 (*n =* 302)	0.95 (0.08; 11.67)	
	55 or over (*n =* 150)	0.75 (0.06; 9.94)	
Ethnicity (*N =* 891)	White (*n =* 790)	Reference	0.51
	Asian (*n =* 51)	3.92 (0.64; 24.06)	
	Black (*n =* 15)	1.22 (0.09; 17.17)	
	Mixed/Other (*n =* 35)	1.59 (0.23; 10.97)	
Region (*N =* 966)	West Midlands (*n =* 58)	Reference	0.02
	East Midlands (*n =* 39)	0.21 (0.02; 2.46)	
	East of England (*n =* 63)	0.45 (0.05; 4.13)	
	London (*n =* 298)	0.66 (0.11; 3.85)	
	Northeast (*n =* 34)	0.08 (0.01; 1.02)	
	Northwest (*n =* 142)	0.04 (0.01; 0.35)	
	Southeast (*n =* 119)	0.23 (0.03; 1.66)	
	Southwest (*n =* 50)	0.11 (0.01; 1.19)	
	Yorkshire and The Humber (*n =* 27)	0.33 (0.02; 5.52)	
	Scotland (*n =* 101)	0.13 (0.02; 1.01)	
	Wales (*n =* 35)	0.01 (0.00; 0.15)	
Type of service (*N =* 978)	Community services (*n =* 834)	Reference	< 0.01
	Crisis services (*n =* 193)	0.25 (0.14; 0.47)	
Profession (*N =* 881)	Clinical or counselling Psychologist (*n =* 322)	Reference	< 0.01
	Nurse (*n =* 276)	0.02 (0.00; 0.11)	
	Occupational Therapist (*n =* 73)	0.05 (0.01; 0.34)	
	Peer Support Worker (*n =* 20)	0.17 (0.01; 2.86)	
	Psychiatrist (*n =* 131)	0.09 (0.02; 0.41)	
	Social Worker (*n =* 59)	0.01 (0.00; 0.10)

Profession, region, and type of service were included in a single analysis model and, when combined, there was still evidence (*p* = 0.05) of telemental health technology use varying by profession (*p* < 0.01) and type of service (*p* < 0.01) (Table 3). Thus, after adjusting for other predictors, use was higher in community settings compared to crisis services. Clinical psychologists and peer support workers used telemental health the most, with other professions using it less. There was a trend towards an overall effect for region, however the overall test was not significant (*p* = 0.07).

**Table 3:** Results of final model

Variable	Value	Odds Ratio (95%CI)	*P* value of overall test
Profession	Clinical Psychologist	reference	< 0.01
	Nurse	0.06 (0.02; 0.23)	
	Occupational Therapist	0.11 (0.03; 0.47)	
	Peer Support Worker	0.36 (0.04; 3.10)	
	Psychiatrist	0.16 (0.05; 0.56)	
	Social Worker	0.03 (0.00; 0.17)	
Region	West Midlands	reference	0.07
	East Midlands	0.25 (0.04; 1.64)	
	East of England	0.46 (0.08; 2.48)	
	London	0.5 (0.13; 1.96)	
	Northeast	0.1 (0.01; 0.78)	
	Northwest	0.1 (0.02; 0.52)	
	Southeast	0.32 (0.07; 1.52)	
	Southwest	0.18 (0.03; 1.2)	
	Yorkshire and The Humber	0.88 (0.10; 7.89)	
	Scotland	0.15 (0.03; 0.80)	
	Wales	0.03 (0.00; 0.24)	
Type of service	Community services	reference	< 0.01
	Crisis services	0.36 (0.18; 0.69)	

### Details of use of telemental health technology

Summaries of responses to survey questions related to staff views of telemental health technologies are presented in Tables 4 and 5. The early stages of the pandemic were associated with a rapid adoption of video and phone technologies for contacting service users and for holding meetings between staff. In community services, just 1.5% (16/1097) of staff reported continuing to meet face-to-face with service users as much as usual. A slight majority (54.8%; 601/1097) reported doing so if strictly necessary, while 42.8% (469/1097) reported stopping face-to-face appointments altogether. Instead, 84.2% (923/1096) reported aiming to replace all cancelled face-to-face appointments with video or phone call appointments, with 13.2% (145/1096) doing so only if appointments could not be postponed. Meanwhile, in crisis settings, 63.1% (166/263) reported only visiting service users at home if strictly necessary, and 5.7% (15/263) reported stopping home visits. 60.6% (157/259) of crisis staff reported having difficulty finding a suitable base for face-to-face meetings.

However, adoption of such technology posed significant challenges for staff. A substantial proportion of clinicians across community and crisis settings reported that technological difficulties with remote appointments were moderately to extremely relevant (crisis: 47.7% (123/258); community: 67.3% (732/1088)). Around half also reported moderate to severe challenges with maintaining engagement with service users (crisis: 54.5% (140/257); community: 65.9% (715/1085)) or assessing them (crisis: 57.8% (148/256); community: 64.1% (695/1085)). In crisis settings specifically, over half (60.6% (155/256)) of staff reported moderate to severe challenges in remotely managing crises at home, while in community settings two-thirds (67.8% (738/1089)) of staff reported similar challenges providing sufficient support to all on their caseloads remotely.

However, more staff also agreed (or strongly agreed) than disagreed that the use of telemental health technologies made it easier to reach some of their clients (crisis: 40.7% (85/209) agreed; community: 42.5% (416/980) agreed), and that they were interested in making more use of such technologies following the end of the COVID-19 pandemic (crisis: 59.0% (125/212) agreed for video and 48.6% (103/212) agreed for phone calls; community: 58.4% (574/983) agreed for video calls and 43.5%; 427/982 agreed for telephone calls). Staff generally felt confident using such technologies to contact their clients (crisis: 45.5% (96/2110); community: 46.4% (452/975)) and typically said that they had the necessary equipment and support to do so (crisis: 58.1% (122/210); community: 58.3% (571/980)). Video calls were generally favoured compared to telephone calls by staff in both community and crisis care for making initial assessments (crisis: 42.3% (91/215) agreed that video calls were a satisfactory method compared with 31.8% (69/217) for telephone calls; community: 39.7% (389/979) for video calls and 30.0% (295/984) for telephone calls) and for assessing the progress of their clients (crisis: 23.3% (156/215) for video calls and 18.5% (161/216) for telephone calls; community: 74.2% (725/977) for video calls and 71.6% (705/985) for telephone calls). It was also believed that establishing a rapport was more challenging by phone compared to video call by staff in both settings (crisis: 33.5% (72/215) agreed that video calls did not substantially affect establishing rapport compared with 26.3% (56/213) for phone calls; community: 34.3% (333/971) for video calls and 27.4% (268/977) for telephone calls).

Video meetings and conference calls were also seen as an acceptable way for staff to meet each other, with video meetings being slightly favoured, and staff viewed using these technologies in such contexts more positively than they did for contacting clients (crisis: 77.7% (167/215) agreed that video calls are a good way of holding meetings compared to 64.0% (137/214) for telephone calls; community: 73.2% (717/979) for video calls and 54.8% (534/974) for telephone calls).

**Table 4:** Survey questions for staff working in community teams and psychological treatment services

Question	Yes, as usual	Yes, if strictly necessary	No	We don’t usually do this	-	-	Total
Are you continuing to visit clients?	21 (1.9%)	557 (50.9%)	336 (30.7%)	181 (16.5%)	-	-	1095
Are you continuing to meet face-to-face with clients?	16 (1.5%)	601 (54.8%)	469 (42.8%)	11 (1.0%)	-	-	1097
**Question**	**Yes, we aim to replace all or almost all cancelled face-to-face appointments with phone or video appointments**	**Yes, for appointments that cannot readily be postponed**	**No, not usually**	-	-	-	**Total**
Are you offering telephone or video call appointments instead of face-to-face appointments?	923 (84.2%)	145 (13.2%)	28 (2.6%)	-	-	-	1096
**Question**	**Yes, aiming to conduct full psychological treatment by phone or video call**	**Yes, but in an abbreviated form**	**No, not usually**	**Not applicable**	-	-	**Total**
Are you offering psychological treatment by phone or by video call as a substitute for face-to-face appointments?	528 (48.4%)	360 (33.0%)	71 (6.5%)	132 (12.1%)	-	-	1091
**How relevant has each of the following challenges been to you at work since mid-March 2020?**	**Not relevant**	**Slightly**	**Moderately**	**Very**	**Extremely relevant**	-	**Total**
Technological difficulties with remote appointments	97 (8.9%)	259 (23.8%)	298 (27.4%)	212 (19.5%)	222 (20.4%)	-	1088
Difficulties engaging clients in remote appointments	106 (9.8%)	264 (24.3%)	310 (28.6%)	207 (19.1%)	198 (18.3%)	-	1085
Difficulty assessing clients by phone or video call	116 (10.7%)	274 (25.3%)	291 (26.8%)	200 (18.4%)	204 (18.8%)	-	1085
Difficulty providing sufficient support with reduced numbers of face-to-face contacts	137 (12.6%)	214 (19.7%)	252 (23.1%)	244 (22.4%)	242 (22.2%)	-	1089
**Remote Appointments**	**Strongly disagree**	**Disagree**	**Neither agree nor disagree**	**Agree**	**Strongly Agree**	**N/A**	**Total**
Telephone calls are often a satisfactory way to make an initial assessment	156 (15.9%)	365 (37.1%)	168 (17.1%)	237 (24.1%)	58 (5.9%)	-	984
Video consultations are often a satisfactory way to way to make an initial assessment	73 (7.5%)	253 (25.8%)	264 (27.0%)	317 (32.4%)	72 (7.4%)	-	979
Telephone calls are often a satisfactory way to assess the progress of someone already known to the team	17 (1.7%)	103 (10.5%)	160 (16.2%)	575 (58.4%)	130 (13.2%)	-	985
Video consultations are often a satisfactory way to assess the progress of someone already known to the team	9 (0.9%)	67 (6.9%)	176 (18.0%)	545 (55.8%)	180 (18.4%)	-	977
Telephone calls are a reasonable way to conduct psychological treatment	110 (11.3%)	296 (30.3%)	239 (24.4%)	223 (22.8%)	42 (4.3%)	68 (7.0%)	978
Video calls are a reasonable way to conduct psychological treatment	57 (5.9%)	150 (15.5%)	264 (27.2%)	343 (35.3%)	80 (8.2%)	77 (7.9%)	971
I hope to meet clients face-to-face just as much as before when the COVID-19 pandemic has finished	21 (2.2%)	131 (13.4%)	128 (13.1%)	328 (33.5%)	371 (37.9%)	-	979
I am interested in making more use of video consultations than previously once the COVID-19 pandemic has finished	75 (7.6%)	164 (16.7%)	170 (17.3%)	403 (41.0%)	171 (17.4%)	-	983
I am interested in making more use of telephone calls than previously once the COVID-19 pandemic is finished	88 (9.0%)	243 (24.8%)	224 (22.8%)	332 (33.8%)	95 (9.7%)	-	982
Using phone rather than face-to-face contact is not too much of a problem for establishing a rapport	184 (18.8%)	361 (37.0%)	164 (16.8%)	214 (21.9%)	54 (5.5%)	-	977
Using video consultation rather than face-to-face contact is not too much of a problem for establishing a rapport	91 (9.4%)	280 (28.8%)	267 (27.5%)	284 (29.3%)	49 (5.1%)	-	971
The clients I see are sometimes easier to reach via phone or video consultation	107 (10.9%)	239 (24.4%)	218 (22.2%)	344 (35.1%)	72 (7.4%)	-	980
Offering remote rather than face-to-face contacts has meant some clients have not been seen	46 (4.7%)	152 (15.5%)	136 (13.9%)	454 (46.4%)	190 (19.4%)	-	978
Email or text messaging is the best way to keep in touch with some of my clients	125 (12.8%)	212 (21.7%)	255 (26.1%)	324 (33.1%)	63 (6.4%)	-	979
I have the necessary equipment and support to be able to carry out video consultations	120 (12.2%)	177 (18.1%)	112 (11.4%)	413 (42.1%)	158 (16.1%)	-	980
The clients I see are generally difficult to engage through phone or video consultations	33 (3.4%)	240 (24.5%)	342 (34.9%)	262 (26.8%)	102 (10.4%)	-	979
I feel confident in using video consultations for client contacts	82 (8.4%)	220 (22.6%)	221 (22.7%)	360 (36.9%)	92 (9.4%)	-	975
Conference calls are a good way of conducting meetings between staff	52 (5.3%)	204 (20.9%)	184 (18.9%)	351 (36.0%)	183 (18.8%)	-	974
Video meetings (e.g. on Microsoft Teams) are a good way of conducting meetings between staff	24 (2.5%)	80 (8.2%)	158 (16.1%)	429 (43.8%)	288 (29.4%)	-	979

**Table 5:** Survey questions for staff working in services offering crisis assessments

Question	Yes, as usual	Yes, if strictly necessary	No	We don’t usually do this		Total
Are you continuing to visit service users at home?	32 (12.2%)	166 (63.1%)	15 (5.7%)	50 (19.0%)		263
**How relevant has each of the following challenges been to you at work since mid-March 2020?**	**Not relevant**	**Slightly**	**Moderately**	**Very**	**Extremely relevant**	**Total**
Lack of a base where clients can be seen face to face	102 (39.4%)	45 (17.4%)	32 (12.4%)	37 (14.3%)	43 (16.6%)	259
Technological difficulties with remote appointments	58 (22.5%)	77 (29.8%)	49 (19.0%)	33 (12.8%)	41 (15.9%)	258
Difficulties engaging clients in remote appointments	48 (18.7%)	69 (26.9%)	64 (24.9%)	39 (15.2%)	37 (14.4%)	257
Difficulty assessing clients by phone or video call	43 (16.8%)	65 (25.4%)	64 (25.0%)	44 (17.2%)	40 (15.6%)	256
Difficulty managing crises at home when no or few face-to-face contacts	53 (20.7%)	48 (18.7%)	62 (24.2%)	53 (20.7%)	40 (15.6%)	256
**Remote Appointments**	**Strongly disagree**	**Disagree**	**Neither agree nor disagree**	**Agree**	**Strongly Agree**	**Total**
Telephone calls are often a satisfactory way to make an initial assessment	31 (14.3%)	75 (34.6%)	42 (19.4%)	59 (27.2%)	10 (4.6%)	217
Video consultations are often a satisfactory way to way to make an initial assessment	12 (5.6%)	45 (20.9%)	67 (31.2%)	74 (34.4%)	17 (7.9%)	215
Telephone calls are often a satisfactory way to assess the progress of someone already known to the team	3 (1.4%)	21 (9.7%)	31 (14.4%)	134 (62.0%)	27 (12.5%)	216
Video consultations are often a satisfactory way to assess the progress of someone already known to the team	1 (0.5%)	15 (7.0%)	43 (20.0%)	117 (54.4%)	39 (18.1%)	215
I hope to meet clients face-to-face just as much as before when the COVID-19 pandemic has finished	7 (3.3%)	36 (17.1%)	29 (13.8%)	65 (31.0%)	73 (34.8%)	210
I am interested in making more use of video consultations than previously once the COVID-19 pandemic has finished	12 (5.7%)	35 (16.5%)	40 (18.9%)	76 (35.9%)	49 (23.1%)	212
I am interested in making more use of telephone calls than previously once the COVID-19 pandemic is finished	8 (3.8%)	43 (20.3%)	58 (27.4%)	75 (35.4%)	28 (13.2%)	212
Using phone rather than face-to-face contact is not too much of a problem for establishing a rapport	36 (16.9%)	69 (32.4%)	52 (24.4%)	45 (21.1%)	11 (5.2%)	213
Using video consultation rather than face-to-face contact is not too much of a problem for establishing a rapport	20 (9.3%)	61 (28.4%)	62 (28.8%)	58 (27.0%)	14 (6.5%)	215
The clients I see are sometimes easier to reach via phone or video consultation	22 (10.5%)	46 (22.0%)	56 (26.8%)	69 (33.0%)	16 (7.7%)	209
Offering remote rather than face-to-face contacts has meant some clients have not been seen	16 (7.6%)	39 (18.5%)	30 (14.2%)	89 (42.2%)	37 (17.5%)	211
Email or text messaging is the best way to keep in touch with some of my clients	35 (16.5%)	46 (21.7%)	58 (27.4%)	62 (29.3%)	11 (5.2%)	212
I have the necessary equipment and support to be able to carry out video consultations	23 (11.0%)	49 (23.3%)	16 (7.6%)	92 (43.8%)	30 (14.3%)	210
The clients I see are generally difficult to engage through phone or video consultations	7 (3.3%)	39 (18.5%)	78 (37.0%)	55 (26.1%)	32 (15.2%)	211
I feel confident in using video consultations for client contacts	22 (10.4%)	44 (20.9%)	49 (23.2%)	72 (34.1%)	24 (11.4%)	211
Conference calls are a good way of conducting meetings between staff	10 (4.7%)	37 (17.3%)	30 (14.0%)	88 (41.1%)	49 (22.9%)	214
Video meetings (e.g. on Microsoft Teams) are a good way of conducting meetings between staff	3 (1.4%)	16 (7.4%)	29 (13.5%)	82 (38.1%)	85 (39.5%)	215

## Discussion

In this paper, we present findings regarding the adoption of telemental health technologies during the early stages of the COVID-19 pandemic from an online survey of clinical staff conducted by the MHPRU in spring 2020, exploring variations in and experiences of telemental health use. Staff reported rapid adoption of such technologies for both contacting service users and for holding meetings between staff. In community and psychological services, virtually all face-to-face contacts with service users stopped unless they were strictly necessary. Instead, a substantial proportion of staff (84.2%) reported replacing their cancelled face-to-face appointments with video and phone call appointments. Patterns of telemental health use varied by service type, region, and profession in the UK. Staff working in community services reported greater use of telemental health compared to services offering crisis care, as did psychologists and peer support workers compared to other mental healthcare professionals, and staff working in the Northwest, Northeast, Scotland and Wales compared to the West Midlands. In general, staff viewed making some use of remote technologies, and especially video calls, positively for both clinical work and meetings between staff, and the majority believed that they had a potential continuing role in practice post-pandemic. In particular, staff viewed such technologies as improving accessibility for some service users. However, at least in the early stages of adoption, there were also significant challenges for staff in community, psychological, and crisis settings related to technological difficulties, as well as engaging, assessing, and supporting or managing some service users. This was particularly true for crisis services, in which two-thirds of staff reported challenges in remotely managing crises at home. There were also challenges in establishing rapport with service users, especially by phone. As reported in our main paper [7], several key themes were identified regarding the adoption of telemental health technologies in the responses to the open-ended items in the survey. These provide helpful context for understanding the findings of the present study. Areas in which remote working worked well include that it was found to be efficient: it allows prompt responses, is convenient, can be done from home, saves travelling time, and is better for the environment; it was also the best option in many contexts during the pandemic, and it allowed services and therapy to keep going during lockdown. Areas for improvement were that, in many cases, the resources available, such as equipment and video calling platforms available to clinicians, were inadequate for the effective use of telemental health; and that some clinicians were concerned about the impact on communication and therapeutic relationships, and also about the potential for digital exclusion.

Our results show that healthcare professionals working in community services used telemental health more than those working in crisis assessment services, suggesting that staff and/or management find its use more appropriate for certain types of service user contact and/or mental health presentation. Appleton et al. [12] reported that telemental health is considered inappropriate for activities that are particularly common in crisis care, such as engaging and assessing new patients, managing medication, and assessing and managing risk and safeguarding issues. In order to be most effective, many of these activities require the clinician to have physical contact with the service user or to see them in their current environment, for example, for physical health assessments, response to risks, and providing medication. Thus, continuing care services may be more readily adapted to telemental health delivery than comprehensive crisis care delivery, which may be more readily used to monitor progress of service users already in contact [7]. However, while challenges to using telemental health in crisis care are greater, we found clear reports from mental health staff that it can be useful and that they intend to carry on using it after the pandemic; it may be that crisis mental health services can operate more successfully online with preparation and carefully designed procedures rather than rapid adoption as an emergency.

Evidence for the overall effect of region on uptake was mixed. There was a statistically significant difference between regions overall in our initial model, but it was only tending towards significance in our final model. Further research is needed to explore this potential issue further. Such regional variations in telemental health uptake may be due to multiple underlying factors that affect the feasibility, availability and value of using this approach, such as variations in the availability of relevant technologies and prevalence of the skills to use them effectively. The Northeast of England has some of the lowest numbers of internet users as well as the poorest coverage of high-speed broadband in the UK, while regions including the Southeast and London have some of the highest - the so-called ‘digital divide’ [37, 38]. Meanwhile, the proportion of the population with basic digital skills, which includes using email and messaging platforms, is lowest in Wales and the Northeast, and again highest in the Southeast and London [37]. This variation is likely to be in part due to geographic factors, such as urbanisation and the presence of major cities in each of the regions [38]. However, while limited internet access in more sparsely populated areas may explain some of the difference, coverage has improved over the last 10 years, and by the end of 2019, 95% of rural areas in the UK had access to internet speeds which should be sufficient to support the use of telemental health, including video calls [38]. In a study of the factors driving digital inequalities across the UK, Blank, Graham, & Calvino [39] found that, after controlling for demographic variables, geographic differences become non-significant. The apparent geographic differences (both regional and urban/rural) could therefore be due to differences in demographic characteristics, most significantly age, education, and occupation – although it is noteworthy that we did not find a similar effect in our own results. Telemental health could potentially be used to address gaps in access to may be particularly effective in are typically located further apart and need to cover larger areas. An example of this is provided by Lindsay et al. [40], who report on an initiative that used video calls to deliver psychotherapy to underserved groups in rural Mississippi, USA. Some of the other benefits of using telemental health are the time saved not having to travel to appointments, and the improved access this provides for some service users, including people with mobility issues or anxiety [18]. However, doing so equitably will require addressing barriers to adoption caused by age, education, and occupation.

A recent meta-analysis found that psychotherapy delivered via video call was as effective as in-person [41]. It should, however, be noted that other studies have concluded that, whilst promising, the current research on video psychotherapy is limited and lacking generalisability [42], and that there are likely to be differences in video compared with face-to-face psychotherapy, for example, that impact how the therapeutic relationship and alliance develops. However, during a crisis such as the COVID-19 pandemic, our finding that psychologists and peer support workers made more use of telemental health compared to other professionals likely reflects the greater feasibility of adopting remote appointments for these professions, compared to the activities of many other clinical roles such as performing assessments, safeguarding, and managing medications [43]. It may also reflect that telephone therapy already played a substantial part of IAPT practice before the pandemic. However, the apparent success in delivering psychological therapies by video call does suggest that relatively complex interactions requiring a strong therapeutic relationship may be possible remotely, despite doubts sometimes expressed about this [18]. It is also noteworthy that peer support workers used telemental health to a similar extent as psychologists, although considerably fewer peer support workers participated in the current survey compared to the other professions. Some caution should therefore be applied to the interpretation of this finding, particularly in the context of our findings elsewhere that some clinicians have expressed concerns in this area. But it is still an encouraging indication that telemental health may be an acceptable method for providing this type of support. The relatively low number of peer support workers is most likely reflective of the low number of such workers currently employed in the UK. However, their numbers have been rising and are set to substantially increase further over the timescale of the NHS Long Term Plan, which sets a target of adding 4730 peer support workers in England by 2023/24 [44], compared to the approximately 862 that were employed in September 2019 [45]. Telemental health may have a role if the NHS is to fully utilise this expanded workforce, but further research, including qualitative interviews with peer support workers with experience of using telemental health, is required to understand how this method of engaging service users can be most effectively implemented. Equally, it is important that those responsible for implementation engage with staff and patients regarding any potentially negative effects of telemental health and respond effectively to any concerns, such as those raised regarding communication and therapeutic relationships. Adequate resourcing, training, and operational procedures, amongst other issues, will be vital to ensure this technology is being used to benefit clinicians and patients, while helping to mitigate any avoidable negative effects it may have.

### Limitations

Our sample was gathered by rapidly disseminating our questionnaire through a range of channels, and so is not representative of those who work in mental health care settings. Staff engaging in online surveys may well be more familiar, skilled, and confident with the technology used in telemental health, so it is possible that surveyed staff may be more likely to adopt such technologies and to view them more positively across several domains than mental health staff overall and that, among non-surveyed staff, the perceived challenges are more severe and widespread than the survey suggests. In contrast to other research findings (e.g. Blank, Graham, & Covino [46]), we did not find any evidence of age, ethnicity, or other demographic characteristics affecting adoption, which may again be attributable to our sample being relatively skilled with digital technologies. The sample may also over-represent people who have strong concerns regarding the adoption of telemental health, or who are motivated to report successful new practices, particularly given the circumstances during the early stages of the COVID-19 pandemic [7]. We only considered crisis and community mental health services, and future research could also explore the adoption of telemental health services in other services, including those aimed at children and adolescents, or older adults.

The number of Black, Asian, and minority ethnic participants in the survey was relatively low compared to the proportion of non-White staff in the NHS workforce, despite our best efforts to increase response rates amongst these groups. London is also over-represented and some other regions, such as Scotland and Wales, under-represented, which may limit how accurately we captured geographical variation [7].

Due to the rapid onset of the pandemic and the shift to remote working, the survey was designed and conducted within a short time frame. Furthermore, given the unprecedented nature of the pandemic, the survey was not based on an established and validated tool. In the absence of previous evidence to guide survey development, assumptions were made about the likely impact of the pandemic and consequent lockdown on services and service users. However, omissions were noted as the study progressed. An important example of this is that, for the survey item we used as our primary outcome in the present study, we asked clinicians about their use of video call technology rather than all forms of telemental health. Our main analysis therefore does not include telephone support and should be interpreted accordingly. Furthermore, to maximise our data capture in a highly pressurised and unprecedented emergency, we chose not to make responding to all survey items obligatory. This resulted in quite a high level of missing data on some survey items, and this should be noted when interpreting our findings.

### Implications for policy

Overall, despite the pandemic requiring that staff adopt telemental health technologies rapidly, with limited central support and time for mental healthcare providers to prepare, they were generally viewed positively by respondents as a method of providing care in certain contexts. Staff saw the potential for these technologies to improve accessibility for some of their service users and viewed them as an effective method for holding at least some meetings between staff. However, there were challenges as well, which potentially would have been ameliorated under less urgent circumstances. Our findings are broadly consistent with those of other research produced by our research group, and suggest that there is a role for telemental health alongside more traditional forms of service delivery and highlight some of its potential benefits in terms of improved access and choice for service users [12, 19]. Telemental health appears to be relatively acceptable and feasible for delivery of psychological therapies or peer support; but, as one would expect, is less used for activities such as clinical assessments, safeguarding, and managing medication, for which being physically present with the service user is often important. Reflecting this, the use of telemental health was more common in community services than it was in crisis care, where face-to-face contact is more often a necessity. The overall acceptability and feasibility of using telemental health amongst psychologists is especially notable given suggestions that it is challenging to form therapeutic relationships by this means [12].

It is notable that we did not find significant divides in the demographics of those adopting telemental health, although, as noted in our limitations section, this could be at least partially attributable to our data collection methods favouring those with a reasonable level of digital skills. As we have discussed elsewhere [19], digital poverty is a significant concern for the implementation of telemental health as it may be experienced by a significant number of service users and lead to digital exclusion for many. Widely available internet infrastructure with minimal barriers to use, such as cost, along with access to devices for making videocalls at home or in suitably private environments, as well as adequate resourcing, training, and strategies for tackling digital illiteracy, are all important in this regard. However, the regional variations for which we found some evidence are notable and potentially concerning. They suggest that the adoption of telemental health may be seeing its own ‘digital divide’, potentially mirroring that which exists for the availability and use of the internet and other digital technologies. There is potential for telemental health to help improve the accessibility and availability of care, such as engaging hard-to-reach or isolated service users. However, if some regions adopt telemental health more than others, this could also have the effect of exacerbating already existing health inequalities and could potentially produce new ones. The adoption of such technology is essential during pandemics where other forms of engagement are greatly reduced. But beyond such contexts, clinicians and those responsible for developing service delivery need to find the right balance between taking up beneficial opportunities to use telemental health, while avoiding reinforcing consequences of digital exclusion. It is desirable that healthcare professionals can deploy these tools where they improve the accessibility and quality of care. The development of future policy should consider how to tackle such challenges, for example, through providing guidance on appropriate technologies to adopt and support for skills development and training of staff.

### Implications for research

This paper reports findings from a broader survey on the impact of the pandemic on mental health services [7], and the number of questions devoted to the use of telemental health was limited. There are therefore likely to be additional relevant topics not addressed by the current study that could fruitfully be explored in future research. Furthermore, the survey was conducted during the early period of the pandemic when staff were still adapting to these new technologies and associated methods of working. Their experience of and increased familiarity with telemental health acquired during the pandemic may have led them to subsequently modify their views regarding the benefits and limitations of such technologies. Further research that takes a longer-term view of clinicians’ attitudes towards and experiences of such technologies, conducted with larger and more representative samples, is needed.

## Supplementary Information

**Additional file 1.**

## Data Availability

The datasets generated and analyzed during the current study are not publicly available because they are being used for additional research by the author research group, but are available from the corresponding author on reasonable request.
