# Supplementary material for: Variations in the uptake of telemental health technologies in community and crisis mental health services during the early pandemic: a survey of mental health professionals in the UK
Source: BMC Psychiatry. 2022 Dec 9;22:776. doi: 10.1186/s12888-022-04385-1 (PMC9733196; doi:10.1186/s12888-022-04385-1)
Supplement: Supplementary file 1 — Additional file 1. [file 12888_2022_4385_MOESM1_ESM.pdf]

FINAL: Mental Health Policy Research Unit: Covid-19 impact on services

Thank you for taking the time to take part in our survey. It is really very much appreciated during this very busy period. **The survey is intended for people who work in mental health care in the UK in any sector or any speciality.**

Our aim is to better understand how the **COVID-19 pandemic is affecting mental health services and the people who may use them.** To make our work practically useful, we are especially interested in what is being done to manage these impacts, both in the **NHS** and the **voluntary sector**, and in what is working well and what less well. This study is being carried out by the NIHR Mental Health Policy Research Unit (MHPRU) for England, whose core team is based at at University College London (UCL) and King's College London. We are a team of independent university-based researchers, whose remit is to make relevant evidence available to policy makers. Our partners include the Centre for Mental Health and the Mental Elf. We will be feeding back results from the study as quickly as possible to policy makers including the Department of Health and Social Care and NHS England, to study participants and to the wider mental health community, with initial results available within a fortnight.

The survey should take around **15 to 20 minutes** to complete (depending how much you write in the open-ended questions) and the responses you provide are fully confidential. If you don’t have time to finish it, please complete as much as you can (responses are stored as you go). Questions can be skipped if necessary. To help us better understand the impact of COVID-19 on mental health care over time, we ask if you are willing to give your email address, so that we can contact you later for some further data collection. You can complete the survey without leaving your email address if you wish. We ask which region you are based in but not which organisation you work for.

As well as sharing our overall findings with policy makers and study participants, we plan to disseminate it as widely as possible to those likely to be able to make use of the information. We will therefore share our findings on the internet and via social media, as well as publishing in scientific journals. However, the **data will be kept fully confidential**, and no publications will include any information that could allow you to be identified. We are also developing a page of helpful resources, and will add to this as information comes in via this survey and other sources. The survey has been developed using UCL Opinio and is hosted on UCL Information Services Division infrastructure. The responses you provide are therefore not processed by any third parties. This study has been approved by the King's College London research ethics committee (MRA-19/20-18372). If you have any queries, please contact Prof Alan Simpson, co-director of the PRU at King’s College London (alan.simpson@kcl.ac.uk) or Prof Sonia Johnson (s.johnson@ucl.ac.uk), Director of the PRU at UCL.

Finally, we would like to take this opportunity to thank you for all your hard work in continuing to provide mental health care during these very challenging times.

By clicking on the ‘Start’ button below, you confirm you have read and understood all of the above text.

Work setting and population

Q1: Which sector do you work in?

- ☐ NHS
- ☐ Voluntary sector
- ☐ Private
- ☐ Social care or other local government
- ☐ Community or user-led organisations

Q2: Which setting do you currently work in?

You may work in more than one of these settings, please select all that apply

- ☐ Hospital inpatient service
- ☐ Crisis house in the community
- ☐ Residential service/ supported housing in the community
- ☐ Service offering crisis assessments (crisis/home treatment teams, liaison teams, AMHP team (Approved Mental Health Professional Teams)
- ☐ Services offering one-to-one mental health care in the community, including community mental health teams of all types, out-patient services, psychological treatment services, Individual Placement and Support services, voluntary services and any other non-crisis services that mainly provide one-to-one contacts
- ☐ Services providing programmes to groups of service users, including day or drop in service, recovery colleges, employment or activity services, or group programme in the community
- ☐ Other (please describe)

If you have chosen "other", please specify:

Q3: Do you work with any of these groups of people or in any of these specialist settings?

You may work with more than one of these population, please select all that apply

- ☐ Adults of working age
- ☐ Older adults with mental health problems and/or dementia
- ☐ Services for people with drug and alcohol problems
- ☐ Children and adolescents
- ☐ Forensic
- ☐ Perinatal

- [ ] People with intellectual disability and/or autism
- [ ] People with eating disorders
- [ ] Other (please describe)

If you have chosen "other", please specify:

Q4: Is this your usual work setting?

- ◯ Yes
- ◯ I am a locum, agency or bank staff member or a trainee without a usual place of work
- ◯ No, I have been redeployed. Please tell us what type of service you usually work in:

If you have chosen "other", please specify:

About you

Q5: Which best describes your profession or role?

- ○ Clinical or counselling psychologist
- ○ Nurse
- ○ Occupational therapist
- ○ Other qualified therapist
- ○ Peer support worker
- ○ Psychiatrist
- ○ Social worker
- ○ Manager in a mental health care service, no mental health professional qualification
- ○ Other worker who has direct contact with people with mental health problems

Q6: Do you have a role as a manager or lead clinician in your service?

○ Yes ○ No

Q7: Are you a trainee or student (pre-MRCPsych psychiatrist, DCLinPsych trainee or student in other profession)?

○ Yes ○ No

Q8: How long in years have you worked in mental health?

Q9: Which country do you work in?Please note: this survey is primarily about the impact of COVID-19 on mental health care in the UK

- ◯ England
- ◯ Northern Ireland
- ◯ Scotland
- ◯ Wales
- ◯ Other (please state which country)

If you have chosen "other", please specify:

Note: if you have NOT answered/chosen item [1] in question 9, skip the following question

Q10: Which region in England?

- ◯ East Midlands
- ◯ East of England
- ◯ London
- ◯ North East
- ◯ North West
- ◯ South East
- ◯ South West
- ◯ West Midlands
- ◯ Yorkshire and The Humber

Note: if you have NOT answered/chosen item [3] in question 9, skip the following question

Q11: Which region in Scotland?

- ◯ Central
- ◯ Glasgow
- ◯ Highlands and Islands
- ◯ Lothian
- ◯ Mid Scotland and Fife
- ◯ North East
- ◯ South
- ◯ West

Note: if you have NOT answered/chosen item [4] in question 9, skip the following question

Q12: Which region in Wales?

- ◯ Mid Wales
- ◯ North
- ◯ South East
- ◯ South West

Q13: Which best describes the locality in which you work?

- ◯ City or town with a population greater than 100,000
- ◯ Town with a population less than 100,000
- ◯ Rural

Current work challenges

How relevant has each of the following challenges been to you at work since mid-March 2020?

Rate from Not very relevant to Extremely relevant. Please try to give a range of scores so that we can see which are the biggest problems.

Q14: Greater workload than usual

- ○ Not relevant
- ○ Slightly
- ○ Moderately
- ○ Very
- ○ Extremely relevant

Q15: Working longer hours than usual

- ○ Not relevant
- ○ Slightly
- ○ Moderately
- ○ Very
- ○ Extremely relevant

Q16: Service users no longer getting an acceptable service due to service reconfiguration because of COVID-19

- ○ Not relevant
- ○ Slightly
- ○ Moderately
- ○ Very
- ○ Extremely relevant

Q17: The risk I or my colleagues could be infected with COVID-19 at work

- ○ Not relevant
- ○ Slightly
- ○ Moderately
- ○ Very
- ○ Extremely relevant

Q18: The risk family and friends may be infected with COVID-19 through me

- ○ Not relevant
- ○ Slightly
- ○ Moderately
- ○ Very
- ○ Extremely relevant

Q19: Having to adapt too quickly to new ways of working

- ○ Not relevant
- ○ Slightly
- ○ Moderately
- ○ Very
- ○ Extremely relevant

Q20: Having to learn to use new technologies too quickly and/or without sufficient training and support

- ◯ Not relevant
- ◯ Slightly
- ◯ Moderately
- ◯ Very
- ◯ Extremely relevant

Q21: Being expected to use new technologies without reliable access to necessary tools and equipment

- ◯ Not relevant
- ◯ Slightly
- ◯ Moderately
- ◯ Very
- ◯ Extremely relevant

Q22: The risk that COVID-19 will spread between service users I'm working with

- ◯ Not relevant
- ◯ Slightly
- ◯ Moderately
- ◯ Very
- ◯ Extremely relevant

Q23: Concern that physical health care received by service users I work with may not be adequate

- ◯ Not relevant
- ◯ Slightly
- ◯ Moderately
- ◯ Very
- ◯ Extremely relevant

Q24: Lack of protective clothing (PPE) and equipment needed for infection control

- ◯ Not relevant
- ◯ Slightly
- ◯ Moderately
- ◯ Very
- ◯ Extremely relevant

Q25: Difficulty putting infection control measures into practice in the setting I work in

- ◯ Not relevant
- ◯ Slightly
- ◯ Moderately
- ◯ Very
- ◯ Extremely relevant

Q26: Problems resulting from lack of access to testing

- ◯ Not relevant
- ◯ Slightly
- ◯ Moderately
- ◯ Very
- ◯ Extremely relevant

Q27: Working in a different setting or with different clinical problems from usual

- ◯ Not relevant
- ◯ Slightly
- ◯ Moderately
- ◯ Very
- ◯ Extremely relevant

Q28: Pressures resulting from the need to support colleagues through the stresses associated with the pandemic

- ◯ Not relevant
- ◯ Slightly
- ◯ Moderately
- ◯ Very
- ◯ Extremely relevant

Q29: Feeling under pressure from managers or colleagues to be less cautious about infection control than I would like

- ◯ Not relevant
- ◯ Slightly
- ◯ Moderately
- ◯ Very
- ◯ Extremely relevant

Q30: Staff shortages (more than is usual in this setting)

- ◯ Not relevant
- ◯ Slightly
- ◯ Moderately
- ◯ Very
- ◯ Extremely relevant

Q31: Not enough of the team I'm working with are permanently employed in this setting (lots of bank/locum and redeployed staff)

- ◯ Not relevant
- ◯ Slightly
- ◯ Moderately
- ◯ Very
- ◯ Extremely relevant

Q32: Having to respond to additional mental health needs that appear to result from COVID-19

- ◯ Not relevant
- ◯ Slightly
- ◯ Moderately
- ◯ Very
- ◯ Extremely relevant

Q33: Safeguarding and other risk management processes cannot be adequately mobilised due to limited social care, legal or police response

- ◯ Not relevant
- ◯ Slightly
- ◯ Moderately
- ◯ Very
- ◯ Extremely relevant

Q34: Pressure to accept redeployment to a setting where I don’t feel happy to work

- ◯ Not relevant
- ◯ Slightly
- ◯ Moderately
- ◯ Very
- ◯ Extremely relevant

Q35: Feeling less able to do my job than usual because my own well-being has suffered through the stresses of the pandemic

- ◯ Not relevant
- ◯ Slightly
- ◯ Moderately
- ◯ Very
- ◯ Extremely relevant

Q36: Increased difficulty managing work-life balance, for example because of loss of childcare

- ◯ Not relevant
- ◯ Slightly
- ◯ Moderately
- ◯ Very
- ◯ Extremely relevant

Q37: Problems commuting safely to work and back

- ◯ Not relevant
- ◯ Slightly
- ◯ Moderately
- ◯ Very
- ◯ Extremely relevant

Service users' and carers' problems during the COVID-19 pandemic

Since mid-March 2020, how relevant do you think each of the following problems are among the service users and carers you are currently in contact with?

Rate from Not very relevant to Extremely relevant.

Q38: Loneliness due to or made worse by social distancing, self-isolation and/or shielding

- ○ Not relevant
- ○ Slightly
- ○ Moderately
- ○ Very
- ○ Extremely relevant

Q39: Lack of access to usual support from NHS mental health services

- ○ Not relevant
- ○ Slightly
- ○ Moderately
- ○ Very
- ○ Extremely relevant

Q40: Lack of access to usual support from other services (primary care, social care, voluntary sector)

- ○ Not relevant
- ○ Slightly
- ○ Moderately
- ○ Very
- ○ Extremely relevant

Q41: Lack of access to medication and to processes for administering and monitoring it

- ○ Not relevant
- ○ Slightly
- ○ Moderately
- ○ Very
- ○ Extremely relevant

Q42: Difficulty engaging with remote appointments by phone or via digital platforms

- ○ Not relevant
- ○ Slightly
- ○ Moderately
- ○ Very
- ○ Extremely relevant

Q43: Lack of access to usual support networks of family and friends

- ○ Not relevant
- ○ Slightly
- ○ Moderately
- ○ Very
- ○ Extremely relevant

Q44: Worries about getting COVID-19 infection

- ○ Not relevant
- ○ Slightly
- ○ Moderately
- ○ Very
- ○ Extremely relevant

Q45: Worries about family getting COVID-19 infection

- ○ Not relevant
- ○ Slightly
- ○ Moderately
- ○ Very
- ○ Extremely relevant

Q46: Difficulty understanding or following current government requirements on social distancing, self-isolation and/or shielding

- ○ Not relevant
- ○ Slightly
- ○ Moderately
- ○ Very
- ○ Extremely relevant

Q47: Problems with police or other authorities because of lack of understanding of /ability to stick to current government requirements

- ○ Not relevant
- ○ Slightly
- ○ Moderately
- ○ Very
- ○ Extremely relevant

Q48: Lack of access to or of equitable provision of physical healthcare for COVID-19

- ○ Not relevant
- ○ Slightly
- ○ Moderately
- ○ Very
- ○ Extremely relevant

Q49: Diminished access to physical health care for problems other than COVID-19

- ○ Not relevant
- ○ Slightly
- ○ Moderately
- ○ Very
- ○ Extremely relevant

Q50: Risk of increased drug and alcohol use or gambling

- ○ Not relevant
- ○ Slightly
- ○ Moderately
- ○ Very
- ○ Extremely relevant

Q51: Increased risk from abusive domestic relationships

◯ Not relevant ◯ Slightly ◯ Moderately ◯ Very
◯ Extremely relevant

Q52: High personal risk of severe consequences of COVID-19 infection (e.g. due to physical health comorbidities)

◯ Not relevant ◯ Slightly ◯ Moderately ◯ Very
◯ Extremely relevant

Q53: Difficulty getting food, money or other basic resources

◯ Not relevant ◯ Slightly ◯ Moderately ◯ Very
◯ Extremely relevant

Q54: Having to stay at home in poor circumstances, or not having a home to go to

◯ Not relevant ◯ Slightly ◯ Moderately ◯ Very
◯ Extremely relevant

Q55: Loss of liberty and rights due to changes in implementation of mental health legislation

◯ Not relevant ◯ Slightly ◯ Moderately ◯ Very
◯ Extremely relevant

Q56: Increase in reliance on family/family tensions

◯ Not relevant ◯ Slightly ◯ Moderately ◯ Very
◯ Extremely relevant

Q57: Increased difficulties for families/carers

◯ Not relevant ◯ Slightly ◯ Moderately ◯ Very
◯ Extremely relevant

Q58: Lack of usual work and activities

◯ Not relevant ◯ Slightly ◯ Moderately ◯ Very
◯ Extremely relevant

Q59: Effects of COVID-19-related trauma

- ◯ Not relevant
- ◯ Slightly
- ◯ Moderately
- ◯ Very
- ◯ Extremely relevant

Q60: Relapse and deterioration in mental health triggered by COVID-19 stresses

- ◯ Not relevant
- ◯ Slightly
- ◯ Moderately
- ◯ Very
- ◯ Extremely relevant

Q61: Are there any groups of clients about whom you are particularly concerned at present? Please tell us about this:

Q62: Are you seeing any mental health problems that seem to arise directly from the current pandemic? If so, please tell us about them:

Sources of help

How important has each of the following been as a source of help and support in doing your work since mid-March 2020?

Rate from Not important at all to Extremely important. Please try to give a range of scores so that we can see what is most helpful.

Q63: Guidance from my employer on managing clinical and safety needs due to COVID-19

◯ Not important at all ◯ Slightly ◯ Moderately ◯ Very
◯ Extremely important

Q64: Guidance disseminated by the NHS or professional bodies

◯ Not important at all ◯ Slightly ◯ Moderately ◯ Very
◯ Extremely important

Q65: Information from the media or social media

◯ Not important at all ◯ Slightly ◯ Moderately ◯ Very
◯ Extremely important

Q66: Support and advice from my manager(s)

◯ Not important at all ◯ Slightly ◯ Moderately ◯ Very
◯ Extremely important

Q67: Support and information from colleagues

◯ Not important at all ◯ Slightly ◯ Moderately ◯ Very
◯ Extremely important

Q68: New initiatives in NHS mental health services

◯ Not important at all ◯ Slightly ◯ Moderately ◯ Very
◯ Extremely important

Q69: The support offered by local volunteers and mutual aid groups

- ◯ Not important at all
- ◯ Slightly
- ◯ Moderately
- ◯ Very
- ◯ Extremely important

Q70: Support and new initiatives from local voluntary sector organisations

- ◯ Not important at all
- ◯ Slightly
- ◯ Moderately
- ◯ Very
- ◯ Extremely important

Q71: National initiatives to support service users and carers, such as helplines and online peer support

- ◯ Not important at all
- ◯ Slightly
- ◯ Moderately
- ◯ Very
- ◯ Extremely important

Q72: Being aware of public support for key workers

- ◯ Not important at all
- ◯ Slightly
- ◯ Moderately
- ◯ Very
- ◯ Extremely important

Q73: Staff well-being initiatives set up during COVID-19 in my workplace

- ◯ Not important at all
- ◯ Slightly
- ◯ Moderately
- ◯ Very
- ◯ Extremely important

Q74: National initiatives to support staff well-being

- ◯ Not important at all
- ◯ Slightly
- ◯ Moderately
- ◯ Very
- ◯ Extremely important

Q75: Adoption of new digital ways of working

- ◯ Not important at all
- ◯ Slightly
- ◯ Moderately
- ◯ Very
- ◯ Extremely important

Q76: Resilience and resourcefulness in adversity among service users and carers

- ◯ Not important at all
- ◯ Slightly
- ◯ Moderately
- ◯ Very
- ◯ Extremely important

Q77: Please tell us about any innovations or initiatives in the service in which you work that have worked well to address COVID-related challenges:

Q78: Please tell us about any resources or guidance on managing the current situation that you think are particularly helpful:

Q79: Has any innovation or change been made in mental health care that you would like to remain in place after the pandemic subsides?

○ Yes ○ No

If yes, please tell us about this

Q80: Is it practical to follow consistently the rules you have been given on infection control at work?

○ Yes ○ No

If not, why not?

Q81: Please tell us about any other guidance or rules that you have received but struggled to put into practice, and why this is:

Q82: Are you particularly concerned about any potential long-term consequences from the current pandemic?

○ Yes ○ No

If yes, please tell us about this

For staff working in or managing inpatient services or crisis houses

Note: if you have NOT answered/chosen at least one of the following items: [1, 2] in question 2, skip the following question

Q83: What type of inpatient service or crisis house do you work in or manage?

You may work in more than one of these settings, please select all that apply

- ☐ General acute ward
- ☐ Psychiatric intensive care unit
- ☐ Hospital based rehabilitation unit
- ☐ Twenty-four hour residential community based rehabilitation unit
- ☐ Secure or forensic ward
- ☐ Older people ward
- ☐ Children and adolescents ward
- ☐ Mother and baby unit
- ☐ Crisis house (residential acute service outside hospital)
- ☐ Other specialist inpatient setting

How relevant has each of the following challenges been to you at work since mid-March 2020?

Rate from Not very relevant to Extremely relevant.

Note: if you have NOT answered/chosen at least one of the following items: [1, 2] in question 2, skip the following question

Q84: Difficulty obtaining relevant advice or arranging assessment by or transfer to general medical services for physically unwell patients/residents

- ◯ Not relevant
- ◯ Slightly
- ◯ Moderately
- ◯ Very
- ◯ Extremely relevant

Note: if you have NOT answered/chosen at least one of the following items: [1, 2] in question 2, skip the following question

Q85: Difficulty maintaining infection control because inpatients/residents are too unwell to follow procedures

- ◯ Not relevant
- ◯ Slightly
- ◯ Moderately
- ◯ Very
- ◯ Extremely relevant

Note: if you have NOT answered/chosen at least one of the following items: [1, 2] in question 2, skip the following question

Q86: Difficulty maintaining infection control because inpatients/residents with confirmed/suspected COVID-19 cannot be effectively segregated from others in this environment

- ◯ Not relevant
- ◯ Slightly
- ◯ Moderately
- ◯ Very
- ◯ Extremely relevant

Note: if you have NOT answered/chosen at least one of the following items: [1, 2] in question 2, skip the following question

Q87: Difficulty discharging inpatients/residents because they will lack usual support from family and friends

- ◯ Not relevant
- ◯ Slightly
- ◯ Moderately
- ◯ Very
- ◯ Extremely relevant

Note: if you have NOT answered/chosen at least one of the following items: [1, 2] in question 2, skip the following question

Q88: Difficulty discharging people because of concerns that they may be an infection risk in their usual home

- ◯ Not relevant
- ◯ Slightly
- ◯ Moderately
- ◯ Very
- ◯ Extremely relevant

Note: if you have NOT answered/chosen at least one of the following items: [1, 2] in question 2, skip the following question

Q89: Difficulty discharging people because services usually available in community are closed or less available

- ◯ Not relevant
- ◯ Slightly
- ◯ Moderately
- ◯ Very
- ◯ Extremely relevant

Note: if you have NOT answered/chosen at least one of the following items: [1, 2] in question 2, skip the following question

Q90: Challenges protecting people at high risk of severe COVID-19 infection adequately

○ Not relevant ○ Slightly ○ Moderately ○ Very
○ Extremely relevant

Note: if you have NOT answered/chosen at least one of the following items: [1, 2] in question 2, skip the following question

Q91: Risk to staff because of lack of PPE (Personal Protective Equipment)

○ Not relevant ○ Slightly ○ Moderately ○ Very
○ Extremely relevant

Note: if you have NOT answered/chosen at least one of the following items: [1, 2] in question 2, skip the following question

Q92: Lack of activities and facilities/increased boredom and agitation during COVID-19 pandemic

○ Not relevant ○ Slightly ○ Moderately ○ Very
○ Extremely relevant

Note: if you have NOT answered/chosen at least one of the following items: [1, 2] in question 2, skip the following question

Q93: Greater difficulty carrying out usual observations and/or managing disturbed behaviour in the context of COVID-19

○ Not relevant ○ Slightly ○ Moderately ○ Very
○ Extremely relevant

Note: if you have NOT answered/chosen at least one of the following items: [1, 2] in question 2, skip the following question

Q94: Difficulty meeting the physical health needs of people with confirmed/suspected COVID-19 on the ward

○ Not relevant ○ Slightly ○ Moderately ○ Very
○ Extremely relevant

Note: if you have NOT answered/chosen at least one of the following items: [1, 2] in question 2, skip the following question

Q95: Reduced access to advocacy and appeal processes under the Mental Health Act

○ Not relevant ○ Slightly ○ Moderately ○ Very
○ Extremely relevant

Note: if you have NOT answered/chosen at least one of the following items: [1, 2] in question 2, skip the following question

Q96: Concerns about deprivation of liberty in enforcing infection control (e.g. by isolating patients in their rooms)

- ◯ Not relevant
- ◯ Slightly
- ◯ Moderately
- ◯ Very
- ◯ Extremely relevant

Note: if you have NOT answered/chosen at least one of the following items: [1, 2] in question 2, skip the following question

Q97: Pressure to discharge more quickly than usual because of the pandemic

- ◯ Not relevant
- ◯ Slightly
- ◯ Moderately
- ◯ Very
- ◯ Extremely relevant

Questions for managers/people with any access to data about service use trends

If you know the answers to any of the following questions, please tell us:

Note: if you have NOT answered/chosen at least one of the following items: [1, 2] in question 2, skip the following question

Q98: What is the current bed occupancy in your hospital or crisis house?

Enter as a fraction. For example, if 13 out of 14 beds are currently occupied, enter '13/14'. Please enter '999' if not sure.

[ ]

Note: if you have NOT answered/chosen at least one of the following items: [1, 2] in question 2, skip the following question

Q99: What percent of inpatients/residents in your service currently have confirmed or suspected COVID-19 infection?

Please enter '999' if not sure

[ ] %

Note: if you have NOT answered/chosen at least one of the following items: [1, 2] in question 2, skip the following question

Q100: Are any of your inpatients/residents currently receiving oxygen in your unit?

Please enter '999' if not sure

○ Yes ○ No ○ Not sure

Note: if you have NOT answered/chosen at least one of the following items: [1, 2] in question 2, skip the following question

Q101: What has happened to your monthly admission rate during the period of the pandemic?

○ Increased by more than 20%
○ Increased by between 11% and 20%
○ Within 10% of usual over the past year
○ Decreased by between 11% and 20%
○ Decreased by more than 20%
○ Not sure

Note: if you have NOT answered/chosen at least one of the following items: [1, 2] in question 2, skip the following question

Q102: What has happened to the rate of compulsory admissions under the Mental Health Act in the setting where you work during the period of the pandemic?

○ Increased by more than 20%
○ Increased by between 11% and 20%
○ Within 10% of usual over the past year
○ Decreased by between 11% and 20%
○ Decreased by more than 20%
○ Not sure
○ Not applicable, we don't accept patients who are sectioned

Note: if you have NOT answered/chosen at least one of the following items: [1, 2] in question 2, skip the following question

Q103: Could we contact you for more exact data? Please give us details of how to do so if so:

For staff working in or managing residential services and supported housing in the community

Note: if you have NOT answered/chosen at least one of the following items: [3] in question 2, skip the following question

Q104: Which of these best describes your service:

- ◯ 24 hour residential service with health professionals in team
- ◯ 24 hour residential service with no health professionals in team
- ◯ Service with staff working on site at least 5 hours a day, not 24 hours
- ◯ Other supported housing scheme (e.g. floating outreach / visiting support)

How relevant has each of the following challenges been to you at work since mid-March 2020?

Rate from Not very relevant to Extremely relevant.

Note: if you have NOT answered/chosen at least one of the following items: [3] in question 2, skip the following question

Q105: Difficulty maintaining infection control because residents don’t understand or are too unwell to follow procedures

- ◯ Not relevant
- ◯ Slightly
- ◯ Moderately
- ◯ Very
- ◯ Extremely relevant

Note: if you have NOT answered/chosen at least one of the following items: [3] in question 2, skip the following question

Q106: Difficulty maintaining infection control because people cannot be effectively segregated from one another in this environment

- ◯ Not relevant
- ◯ Slightly
- ◯ Moderately
- ◯ Very
- ◯ Extremely relevant

Note: if you have NOT answered/chosen at least one of the following items: [3] in question 2, skip the following question

Q107: Lack of support because of closure of or reduction in community mental health services

- ◯ Not relevant
- ◯ Slightly
- ◯ Moderately
- ◯ Very
- ◯ Extremely relevant

Note: if you have NOT answered/chosen at least one of the following items: [3] in question 2, skip the following question

Q108: Lack of support due to reduction in other services in the community e.g. primary care, social care, voluntary sector services

- ◯ Not relevant
- ◯ Slightly
- ◯ Moderately
- ◯ Very
- ◯ Extremely relevant

Note: if you have NOT answered/chosen at least one of the following items: [3] in question 2, skip the following question

Q109: More challenging environment because residents cannot go out and engage in activities as usual

- ◯ Not relevant
- ◯ Slightly
- ◯ Moderately
- ◯ Very
- ◯ Extremely relevant

Note: if you have NOT answered/chosen at least one of the following items: [3] in question 2, skip the following question

Q110: Difficulty managing communal areas of accommodation safely

- ◯ Not relevant
- ◯ Slightly
- ◯ Moderately
- ◯ Very
- ◯ Extremely relevant

Note: if you have NOT answered/chosen at least one of the following items: [3] in question 2, skip the following question

Q111: Lack of confidence and support in managing residents who are ill with COVID-19 infections

○ Not relevant ○ Slightly ○ Moderately ○ Very
○ Extremely relevant

Note: if you have NOT answered/chosen at least one of the following items: [3] in question 2, skip the following question

Q112: Difficulty getting appropriate medical care for residents who are ill with COVID-19 infections

○ Not relevant ○ Slightly ○ Moderately ○ Very
○ Extremely relevant

Note: if you have NOT answered/chosen at least one of the following items: [3] in question 2, skip the following question

Q113: Challenges supporting residents who are very worried about COVID-19 infection

○ Not relevant ○ Slightly ○ Moderately ○ Very
○ Extremely relevant

Note: if you have NOT answered/chosen at least one of the following items: [3] in question 2, skip the following question

Q114: Not being able to have as much contact as usual with residents due to staff shortages or changes in service offered

○ Not relevant ○ Slightly ○ Moderately ○ Very
○ Extremely relevant

Note: if you have NOT answered/chosen at least one of the following items: [3] in question 2, skip the following question

Q115: Not being able to have as much contact as usual with residents due to quarantine precautions

○ Not relevant ○ Slightly ○ Moderately ○ Very
○ Extremely relevant

Note: if you have NOT answered/chosen at least one of the following items: [3] in question 2, skip the following question

Q116: Has your service had to ask any residents to leave because of concerns about infection control?

○ Yes ○ No

Note: if you have NOT answered/chosen at least one of the following items: [3] in question 2, skip the following question

Q117: Has the availability or nature of your service, or its operating hours changed in any way?

○ Yes ○ No

If yes, please tell us about this

For staff working in or managing services offering crisis assessments

Note: if you have NOT answered/chosen at least one of the following items: [4] in question 2, skip the following question

Q118: Which of the following type(s) of service do you work in?

- ☐ Crisis team/home treatment team/crisis assessment team
- ☐ Crisis phone line
- ☐ Liaison service or decision unit in general hospital
- ☐ Approved Mental Health Professional (AMHP) team
- ☐ Crisis centre or clinic offering assessments
- ☐ Other crisis service (please describe):

If you have chosen "other", please specify:

Note: if you have NOT answered/chosen at least one of the following items: [4] in question 2, skip the following question

Q119: Have any changes occurred to your service’s hours and location?

○ Yes ○ No

If yes, please tell us about this

Note: if you have NOT answered/chosen at least one of the following items: [4] in question 2, skip the following question

Q120: Are you continuing to visit service users at home?

○ Yes, as usual ○ Yes, if strictly necessary ○ No
○ We don't usually do this

Note: if you have NOT answered/chosen at least one of the following items: [4] in question 2, skip the following question

Q121: Has any new form of crisis service been established to meet local needs during the COVID-19 pandemic?

○ Yes ○ No

If yes, please tell us about this

Note: if you have NOT answered/chosen at least one of the following items: [4] in question 2, skip the following question

Q122: How effectively do you feel crises are being managed in your area compared with usual?

- ○ Much less effectively
- ○ Somewhat less
- ○ About the same
- ○ More effectively
- ○ N/A

How relevant has each of the following challenges been to you at work since mid-March 2020?

Rate from Not very relevant to Extremely relevant.

Note: if you have NOT answered/chosen at least one of the following items: [4] in question 2, skip the following question

Q123: Increased demand for crisis assessments

- ◯ Not relevant
- ◯ Slightly
- ◯ Moderately
- ◯ Very
- ◯ Extremely relevant

Note: if you have NOT answered/chosen at least one of the following items: [4] in question 2, skip the following question

Q124: Lack of facility to continue mental health service safely in A and E departments

- ◯ Not relevant
- ◯ Slightly
- ◯ Moderately
- ◯ Very
- ◯ Extremely relevant

Note: if you have NOT answered/chosen at least one of the following items: [4] in question 2, skip the following question

Q125: Difficulty providing a good service for people who have harmed themselves

- ◯ Not relevant
- ◯ Slightly
- ◯ Moderately
- ◯ Very
- ◯ Extremely relevant

Note: if you have NOT answered/chosen at least one of the following items: [4] in question 2, skip the following question

Q126: Lack of a base where clients can be seen face to face

- ◯ Not relevant
- ◯ Slightly
- ◯ Moderately
- ◯ Very
- ◯ Extremely relevant

Note: if you have NOT answered/chosen at least one of the following items: [4] in question 2, skip the following question

Q127: Technological difficulties with remote appointments

- ◯ Not relevant
- ◯ Slightly
- ◯ Moderately
- ◯ Very
- ◯ Extremely relevant

Note: if you have NOT answered/chosen at least one of the following items: [4] in question 2, skip the following question

Q128: Difficulties engaging clients in remote appointments

- ◯ Not relevant
- ◯ Slightly
- ◯ Moderately
- ◯ Very
- ◯ Extremely relevant

Note: if you have NOT answered/chosen at least one of the following items: [4] in question 2, skip the following question

Q129: Difficulty assessing clients by phone or video call

- ○ Not relevant
- ○ Slightly
- ○ Moderately
- ○ Very
- ○ Extremely relevant

Note: if you have NOT answered/chosen at least one of the following items: [4] in question 2, skip the following question

Q130: Difficulty managing crises at home when no or few face to face contacts

- ○ Not relevant
- ○ Slightly
- ○ Moderately
- ○ Very
- ○ Extremely relevant

Note: if you have NOT answered/chosen at least one of the following items: [4] in question 2, skip the following question

Q131: Problems ensuring clients have medication and that it is monitored regularly

- ○ Not relevant
- ○ Slightly
- ○ Moderately
- ○ Very
- ○ Extremely relevant

Note: if you have NOT answered/chosen at least one of the following items: [4] in question 2, skip the following question

Q132: Lack of continuing care from community mental health services

- ○ Not relevant
- ○ Slightly
- ○ Moderately
- ○ Very
- ○ Extremely relevant

Note: if you have NOT answered/chosen at least one of the following items: [4] in question 2, skip the following question

Q133: Not being able to signpost or refer to other services in your area (primary care, social care, voluntary sector services)

- ○ Not relevant
- ○ Slightly
- ○ Moderately
- ○ Very
- ○ Extremely relevant

Note: if you have NOT answered/chosen at least one of the following items: [4] in question 2, skip the following question

Q134: Increased practical difficulties in organising Mental Health Act assessments

- ○ Not relevant
- ○ Slightly
- ○ Moderately
- ○ Very
- ○ Extremely relevant

Note: if you have NOT answered/chosen at least one of the following items: [4] in question 2, skip the following question

Q135: Limited access to general hospital to provide liaison services

- ◯ Not relevant
- ◯ Slightly
- ◯ Moderately
- ◯ Very
- ◯ Extremely relevant

Remote appointments

Note: if you have NOT answered/chosen at least one of the following items: [4] in question 2, skip the following question

Q136: Are you using telephone calls or video consultations (including WhatsApp video, Zoom, Microsoft Teams or any other video call platform) to replace some or all face to face meetings with clients?

◯ Yes ◯ No

Remote appointments (cont.)

To what extent do you agree with the following?

Please rate from Strongly disagree to Strongly agree.

Note: if you have NOT answered/chosen item [1] in question 136, skip the following question

Q137: Telephone calls are often a satisfactory way to make an initial assessment

- ○ Strongly disagree
- ○ Disagree
- ○ Neither agree or disagree
- ○ Agree
- ○ Strongly agree

Note: if you have NOT answered/chosen item [1] in question 136, skip the following question

Q138: Video consultations are often a satisfactory way to way to make an initial assessment

- ○ Strongly disagree
- ○ Disagree
- ○ Neither agree or disagree
- ○ Agree
- ○ Strongly agree

Note: if you have NOT answered/chosen item [1] in question 136, skip the following question

Q139: Telephone calls are often a satisfactory way to assess the progress of someone already known to the team

- ○ Strongly disagree
- ○ Disagree
- ○ Neither agree or disagree
- ○ Agree
- ○ Strongly agree

Note: if you have NOT answered/chosen item [1] in question 136, skip the following question

Q140: Video consultations are often a satisfactory way to assess the progress of someone already known to the team

- ○ Strongly disagree
- ○ Disagree
- ○ Neither agree or disagree
- ○ Agree
- ○ Strongly agree

Note: if you have NOT answered/chosen item [1] in question 136, skip the following question

Q141: I hope to meet clients face to face just as much as before when the COVID-19 pandemic has finished

- ○ Strongly disagree
- ○ Disagree
- ○ Neither agree or disagree
- ○ Agree
- ○ Strongly agree

Note: if you have NOT answered/chosen item [1] in question 136, skip the following question

Q142: I am interested in making more use of video consultations than previously once the COVID-19 pandemic has finished

- ○ Strongly disagree
- ○ Disagree
- ○ Neither agree or disagree
- ○ Agree
- ○ Strongly agree

Note: if you have NOT answered/chosen item [1] in question 136, skip the following question

Q143: I am interested in making more use of telephone calls than previously once the COVID-19 pandemic is finished

- ◯ Strongly disagree
- ◯ Disagree
- ◯ Neither agree or disagree
- ◯ Agree
- ◯ Strongly agree

Note: if you have NOT answered/chosen item [1] in question 136, skip the following question

Q144: Using phone rather than face to face contact is not too much of a problem for establishing a rapport

- ◯ Strongly disagree
- ◯ Disagree
- ◯ Neither agree or disagree
- ◯ Agree
- ◯ Strongly agree

Note: if you have NOT answered/chosen item [1] in question 136, skip the following question

Q145: Using video consultation rather than face to face contact is not too much of a problem for establishing a rapport

- ◯ Strongly disagree
- ◯ Disagree
- ◯ Neither agree or disagree
- ◯ Agree
- ◯ Strongly agree

Note: if you have NOT answered/chosen item [1] in question 136, skip the following question

Q146: The clients I see are sometimes easier to reach via phone or video consultation

- ◯ Strongly disagree
- ◯ Disagree
- ◯ Neither agree or disagree
- ◯ Agree
- ◯ Strongly agree

Note: if you have NOT answered/chosen item [1] in question 136, skip the following question

Q147: Offering remote rather than face-to-face contacts has meant some clients have not been seen

- ◯ Strongly disagree
- ◯ Disagree
- ◯ Neither agree or disagree
- ◯ Agree
- ◯ Strongly agree

Note: if you have NOT answered/chosen item [1] in question 136, skip the following question

Q148: Email or text messaging is the best way to keep in touch with some of my clients

- ◯ Strongly disagree
- ◯ Disagree
- ◯ Neither agree or disagree
- ◯ Agree
- ◯ Strongly agree

Note: if you have NOT answered/chosen item [1] in question 136, skip the following question

Q149: I have the necessary equipment and support to be able to carry out video consultations

- ◯ Strongly disagree
- ◯ Disagree
- ◯ Neither agree or disagree
- ◯ Agree
- ◯ Strongly agree

Note: if you have NOT answered/chosen item [1] in question 136, skip the following question

Q150: The clients I see are generally difficult to engage through phone or video consultations

- ◯ Strongly disagree
- ◯ Disagree
- ◯ Neither agree or disagree
- ◯ Agree
- ◯ Strongly agree

Note: if you have NOT answered/chosen item [1] in question 136, skip the following question

Q151: I feel confident in using video consultations for client contacts

- ◯ Strongly disagree
- ◯ Disagree
- ◯ Neither agree or disagree
- ◯ Agree
- ◯ Strongly agree

Note: if you have NOT answered/chosen item [1] in question 136, skip the following question

Q152: Conference calls are a good way of conducting meetings between staff

- ◯ Strongly disagree
- ◯ Disagree
- ◯ Neither agree or disagree
- ◯ Agree
- ◯ Strongly agree

Note: if you have NOT answered/chosen item [1] in question 136, skip the following question

Q153: Video meetings (e.g. on Microsoft Teams) are a good way of conducting meetings between staff

- ◯ Strongly disagree
- ◯ Disagree
- ◯ Neither agree or disagree
- ◯ Agree
- ◯ Strongly agree

Note: if you have NOT answered/chosen item [1] in question 136, skip the following question

Q154: What, if any, way of conducting remote appointments is working well for you?

Note: if you have NOT answered/chosen item [1] in question 136, skip the following question

Q155: What, if any, way of conducting remote appointments has not worked well for you and why?

Note: if you have NOT answered/chosen item [1] in question 136, skip the following question

Q156: Please give an estimate of the percentage of clients you see with whom you now mainly have contact by video call

%

Questions for managers/people with any access to data about service use trends

If you know the answers to any of the following questions, please tell us:

Note: if you have NOT answered/chosen at least one of the following items: [4] in question 2, skip the following question

Q157: What has happened to the size of your team's caseload currently compared with the team's typical caseload?

- ◯ Increased by more than 20%
- ◯ Increased by between 11% and 20%
- ◯ Within 10% of usual over the past year
- ◯ Decreased by between 11% and 20%
- ◯ Decreased by more than 20%
- ◯ Not sure

Note: if you have NOT answered/chosen at least one of the following items: [4] in question 2, skip the following question

Q158: What has happened to the rate of referrals to your service?

- ◯ Increased by more than 20%
- ◯ Increased by between 11% and 20%
- ◯ Within 10% of usual over the past year
- ◯ Decreased by between 11% and 20%
- ◯ Decreased by more than 20%
- ◯ Not sure

Note: if you have NOT answered/chosen at least one of the following items: [4] in question 2, skip the following question

Q159: Could we contact you for more exact data? Please give us details of how to do so if so:

For staff working in or managing community teams and psychological treatment services

Note: if you have NOT answered/chosen at least one of the following items: [5] in question 2, skip the following question

Q160: Which of the following type(s) of service do you work in?

- ☐ Early intervention service
- ☐ Assertive outreach or rehabilitation team
- ☐ Other type of multidisciplinary community mental health team
- ☐ Improved Access to Psychological Treatments (IAPT) service
- ☐ Other psychological treatment or therapy service
- ☐ Individual Placement and Support (IPS) service
- ☐ Primary care mental health service
- ☐ Memory service
- ☐ Voluntary sector support service that mainly works one-to-one with clients
- ☐ Other, please describe:

If you have chosen "other", please specify:

Note: if you have NOT answered/chosen at least one of the following items: [5] in question 2, skip the following question

Q161: Have any changes occurred to your service's hours and location?

○ Yes ○ No

If yes, please tell us about this

Note: if you have NOT answered/chosen at least one of the following items: [5] in question 2, skip the following question

Q162: Are you continuing to visit clients?

○ Yes, as usual ○ Yes, if strictly necessary ○ No
○ We don't usually do this

Note: if you have NOT answered/chosen at least one of the following items: [5] in question 2, skip the following question

Q163: Are you continuing to meet face to face with clients?

- ◯ Yes, as usual
- ◯ Yes, if strictly necessary
- ◯ No
- ◯ We don't usually do this

Note: if you have NOT answered/chosen at least one of the following items: [5] in question 2, skip the following question

Q164: Are you offering telephone or video call appointments instead of face to face appointments?

- ◯ Yes, we aim to replace all or almost all cancelled face to face appointments with phone or video appointments
- ◯ Yes, for appointments that cannot readily be postponed
- ◯ No, not usually

Note: if you have NOT answered/chosen at least one of the following items: [5] in question 2, skip the following question

Q165: Are you offering psychological treatment by phone or by video call as a substitute for face to face appointments?

- ◯ Yes, aiming to conduct full psychological treatment by phone or video call
- ◯ Yes, but in an abbreviated form
- ◯ No, not usually
- ◯ Not applicable

Note: if you have NOT answered/chosen at least one of the following items: [5] in question 2, skip the following question

Q166: Have you started making more use of digital tools for assessment or treatment (internet-based or apps) as a substitute for face to face approaches?

- ◯ Yes
- ◯ No

If yes, what have you used and how has it gone?

Note: if you have NOT answered/chosen at least one of the following items: [5] in question 2, skip the following question

Q167: Is your service providing any new type of help in response to the COVID-19 crisis?

- ◯ Yes
- ◯ No

If yes, please tell us about this

How relevant has each of the following challenges been to you at work since mid-March 2020?

Rate from Not very relevant to Extremely relevant.

Note: if you have NOT answered/chosen at least one of the following items: [5] in question 2, skip the following question

Q168: Increased pressure due to clients deteriorating

- ○ Not relevant
- ○ Slightly
- ○ Moderately
- ○ Very
- ○ Extremely relevant

Note: if you have NOT answered/chosen at least one of the following items: [5] in question 2, skip the following question

Q169: Increased demand due to large numbers of referrals

- ○ Not relevant
- ○ Slightly
- ○ Moderately
- ○ Very
- ○ Extremely relevant

Note: if you have NOT answered/chosen at least one of the following items: [5] in question 2, skip the following question

Q170: Increased pressure because of our clients' practical and social problems, such as access to food and money

- ○ Not relevant
- ○ Slightly
- ○ Moderately
- ○ Very
- ○ Extremely relevant

Note: if you have NOT answered/chosen at least one of the following items: [5] in question 2, skip the following question

Q171: Technological difficulties with remote appointments

- ○ Not relevant
- ○ Slightly
- ○ Moderately
- ○ Very
- ○ Extremely relevant

Note: if you have NOT answered/chosen at least one of the following items: [5] in question 2, skip the following question

Q172: Difficulties engaging clients in remote appointments

- ○ Not relevant
- ○ Slightly
- ○ Moderately
- ○ Very
- ○ Extremely relevant

Note: if you have NOT answered/chosen at least one of the following items: [5] in question 2, skip the following question

Q173: Difficulty assessing clients by phone or video call

- ○ Not relevant
- ○ Slightly
- ○ Moderately
- ○ Very
- ○ Extremely relevant

Note: if you have NOT answered/chosen at least one of the following items: [5] in question 2, skip the following question

Q174: Difficulty providing sufficient support with reduced numbers of face to face contacts

- ○ Not relevant
- ○ Slightly
- ○ Moderately
- ○ Very
- ○ Extremely relevant

Note: if you have NOT answered/chosen at least one of the following items: [5] in question 2, skip the following question

Q175: Problems ensuring clients have medication

- ○ Not relevant
- ○ Slightly
- ○ Moderately
- ○ Very
- ○ Extremely relevant

Note: if you have NOT answered/chosen at least one of the following items: [5] in question 2, skip the following question

Q176: Problems ensuring safe continuation of medication that requires administration or monitoring in person e.g. depots, clozapine, lithium

- ○ Not relevant
- ○ Slightly
- ○ Moderately
- ○ Very
- ○ Extremely relevant

Note: if you have NOT answered/chosen at least one of the following items: [5] in question 2, skip the following question

Q177: Not being able to depend on other services that are normally available in the community (primary care, social care, voluntary sector services)

- ○ Not relevant
- ○ Slightly
- ○ Moderately
- ○ Very
- ○ Extremely relevant

Note: if you have NOT answered/chosen at least one of the following items: [5] in question 2, skip the following question

Q178: Increased practical difficulties in organising Mental Health Act assessments

- ○ Not relevant
- ○ Slightly
- ○ Moderately
- ○ Very
- ○ Extremely relevant

Note: if you have NOT answered/chosen at least one of the following items: [5] in question 2, skip the following question

Q179: Less access than usual to crisis care outside hospital

- ○ Not relevant
- ○ Slightly
- ○ Moderately
- ○ Very
- ○ Extremely relevant

Note: if you have NOT answered/chosen at least one of the following items: [5] in question 2, skip the following question

Q180: Greater difficulty than usual in arranging hospital admission

- ◯ Not relevant
- ◯ Slightly
- ◯ Moderately
- ◯ Very
- ◯ Extremely relevant

Note: if you have NOT answered/chosen at least one of the following items: [5] in question 2, skip the following question

Q181: Our reluctance to admit clients to psychiatric hospital because of COVID-19 risk

- ◯ Not relevant
- ◯ Slightly
- ◯ Moderately
- ◯ Very
- ◯ Extremely relevant

Note: if you have NOT answered/chosen at least one of the following items: [5] in question 2, skip the following question

Q182: Challenges supporting clients in residential settings (e.g. due to infection control challenges)

- ◯ Not relevant
- ◯ Slightly
- ◯ Moderately
- ◯ Very
- ◯ Extremely relevant

Note: if you have NOT answered/chosen at least one of the following items: [5] in question 2, skip the following question

Q183: Increased work to engage and support homeless people in the area

- ◯ Not relevant
- ◯ Slightly
- ◯ Moderately
- ◯ Very
- ◯ Extremely relevant

Note: if you have NOT answered/chosen at least one of the following items: [5] in question 2, skip the following question

Q184: Have you managed any clients at home because of concerns about COVID-19 risk who would usually have been admitted to hospital?

◯ Yes ◯ No

Remote appointments

Note: if you have NOT answered/chosen at least one of the following items: [5] in question 2, skip the following question

Q185: Are you using telephone calls or video consultations (including WhatsApp video, Zoom, Microsoft Teams or any other video call platform) to replace some or all face to face meetings with clients?

◯ Yes ◯ No

Remote appointments (cont.)

To what extent do you agree with the following?

Please rate from Strongly disagree to Strongly agree.

Note: if you have NOT answered/chosen item [1] in question 185, skip the following question

Q186: Telephone calls are often a satisfactory way to make an initial assessment

- ◯ Strongly disagree
- ◯ Disagree
- ◯ Neither agree or disagree
- ◯ Agree
- ◯ Strongly agree

Note: if you have NOT answered/chosen item [1] in question 185, skip the following question

Q187: Video consultations are often a satisfactory way to way to make an initial assessment

- ◯ Strongly disagree
- ◯ Disagree
- ◯ Neither agree or disagree
- ◯ Agree
- ◯ Strongly agree

Note: if you have NOT answered/chosen item [1] in question 185, skip the following question

Q188: Telephone calls are often a satisfactory way to assess the progress of someone already known to the team

- ◯ Strongly disagree
- ◯ Disagree
- ◯ Neither agree or disagree
- ◯ Agree
- ◯ Strongly agree

Note: if you have NOT answered/chosen item [1] in question 185, skip the following question

Q189: Video consultations are often a satisfactory way to assess the progress of someone already known to the team

- ◯ Strongly disagree
- ◯ Disagree
- ◯ Neither agree or disagree
- ◯ Agree
- ◯ Strongly agree

Note: if you have NOT answered/chosen item [1] in question 185, skip the following question

Q190: Telephone calls are a reasonable way to conduct psychological treatment

- ◯ Strongly disagree
- ◯ Disagree
- ◯ Neither agree or disagree
- ◯ Agree
- ◯ Strongly agree
- ◯ N/A

Note: if you have NOT answered/chosen item [1] in question 185, skip the following question

Q191: Video calls are a reasonable way to conduct psychological treatment

- ◯ Strongly disagree
- ◯ Disagree
- ◯ Neither agree or disagree
- ◯ Agree
- ◯ Strongly agree
- ◯ N/A

Note: if you have NOT answered/chosen item [1] in question 185, skip the following question

Q192: I hope to meet clients face to face just as much as before when the COVID-19 pandemic has finished

- ◯ Strongly disagree
- ◯ Disagree
- ◯ Neither agree or disagree
- ◯ Agree
- ◯ Strongly agree

Note: if you have NOT answered/chosen item [1] in question 185, skip the following question

Q193: I am interested in making more use of video consultations than previously once the COVID-19 pandemic has finished

- ◯ Strongly disagree
- ◯ Disagree
- ◯ Neither agree or disagree
- ◯ Agree
- ◯ Strongly agree

Note: if you have NOT answered/chosen item [1] in question 185, skip the following question

Q194: I am interested in making more use of telephone calls than previously once the COVID-19 pandemic is finished

- ◯ Strongly disagree
- ◯ Disagree
- ◯ Neither agree or disagree
- ◯ Agree
- ◯ Strongly agree

Note: if you have NOT answered/chosen item [1] in question 185, skip the following question

Q195: Using phone rather than face to face contact is not too much of a problem for establishing a rapport

- ◯ Strongly disagree
- ◯ Disagree
- ◯ Neither agree or disagree
- ◯ Agree
- ◯ Strongly agree

Note: if you have NOT answered/chosen item [1] in question 185, skip the following question

Q196: Using video consultation rather than face to face contact is not too much of a problem for establishing a rapport

- ◯ Strongly disagree
- ◯ Disagree
- ◯ Neither agree or disagree
- ◯ Agree
- ◯ Strongly agree

Note: if you have NOT answered/chosen item [1] in question 185, skip the following question

Q197: The clients I see are sometimes easier to reach via phone or video consultation

- ◯ Strongly disagree
- ◯ Disagree
- ◯ Neither agree or disagree
- ◯ Agree
- ◯ Strongly agree

Note: if you have NOT answered/chosen item [1] in question 185, skip the following question

Q198: Offering remote rather than face-to-face contacts has meant some clients have not been seen

- Strongly disagree
- Disagree
- Neither agree or disagree
- Agree
- Strongly agree

Note: if you have NOT answered/chosen item [1] in question 185, skip the following question

Q199: Email or text messaging is the best way to keep in touch with some of my clients

- Strongly disagree
- Disagree
- Neither agree or disagree
- Agree
- Strongly agree

Note: if you have NOT answered/chosen item [1] in question 185, skip the following question

Q200: I have the necessary equipment and support to be able to carry out video consultations

- Strongly disagree
- Disagree
- Neither agree or disagree
- Agree
- Strongly agree

Note: if you have NOT answered/chosen item [1] in question 185, skip the following question

Q201: The clients I see are generally difficult to engage through phone or video consultations

- Strongly disagree
- Disagree
- Neither agree or disagree
- Agree
- Strongly agree

Note: if you have NOT answered/chosen item [1] in question 185, skip the following question

Q202: I feel confident in using video consultations for client contacts

- Strongly disagree
- Disagree
- Neither agree or disagree
- Agree
- Strongly agree

Note: if you have NOT answered/chosen item [1] in question 185, skip the following question

Q203: Conference calls are a good way of conducting meetings between staff

- Strongly disagree
- Disagree
- Neither agree or disagree
- Agree
- Strongly agree

Note: if you have NOT answered/chosen item [1] in question 185, skip the following question

Q204: Video meetings (e.g. on Microsoft Teams) are a good way of conducting meetings between staff

- Strongly disagree
- Disagree
- Neither agree or disagree
- Agree
- Strongly agree

Note: if you have NOT answered/chosen item [1] in question 185, skip the following question

Q205: What, if any, way of conducting remote appointments is working well for you?

Note: if you have NOT answered/chosen item [1] in question 185, skip the following question

Q206: What, if any, way of conducting remote appointments has not worked well for you and why?

Note: if you have NOT answered/chosen item [1] in question 185, skip the following question

Q207: Please give an estimate of the percentage of clients you see with whom you now mainly have contact by video call?

%

Note: if you have NOT answered/chosen at least one of the following items: [5] in question 2, skip the following question

Q208: What has happened to the rate of referrals to your team during the period of the pandemic?

- ◯ Increased by more than 20%
- ◯ Increased by between 11% and 20%
- ◯ Within 10% of usual over the past year
- ◯ Decreased by between 11% and 20%
- ◯ Decreased by more than 20%
- ◯ Not sure

Note: if you have NOT answered/chosen at least one of the following items: [5] in question 2, skip the following question

Q209: What has happened to the number of client contacts you have each week?

- ◯ Increased by more than 20%
- ◯ Increased by between 11% and 20%
- ◯ Within 10% of usual over the past year
- ◯ Decreased by between 11% and 20%
- ◯ Decreased by more than 20%
- ◯ Not sure

For staff working in or managing: Day centres, Drop-in services, Social firms, Group programmes, Recovery colleges, Crisis cafes, Clubhouses

Note: if you have NOT answered/chosen at least one of the following items: [6] in question 2, skip the following question

Q210: What type(s) of service do you work in?

- [ ] Day centre
- [ ] Drop-in service
- [ ] Social firm
- [ ] Group programme
- [ ] Recovery college
- [ ] Crisis cafe
- [ ] Clubhouse
- [ ] Other, please describe

If you have chosen "other", please specify:

Note: if you have NOT answered/chosen at least one of the following items: [6] in question 2, skip the following question

Q211: Are you providing any online group programme during the COVID-19 crisis?

- ○ Yes
- ○ No

If yes, who is it for?

Note: if you have NOT answered/chosen at least one of the following items: [6] in question 2, skip the following question

Q212: Is it

- ○ Local
- ○ National
- ○ Not applicable

Note: if you have NOT answered/chosen at least one of the following items: [6] in question 2, skip the following question

Q213: What if anything has worked well?

Note: if you have NOT answered/chosen at least one of the following items: [6] in question 2, skip the following question

Q214: What has not worked well?

Note: if you have NOT answered/chosen at least one of the following items: [6] in question 2, skip the following question

Q215: Are you continuing any other service or client contact during the current COVID-19 pandemic?

◯ Yes ◯ No

If yes, please tell us what this involves and how it has gone

For staff working in or managing older adult services (including dementia)

How relevant has each of the following challenges been to you at work since mid-March 2020?

Rate from Not very relevant to Extremely relevant.

Note: if you have NOT answered/chosen at least one of the following items: [2] in question 3, skip the following question

Q216: Challenges supporting clients and staff in nursing homes and care homes where there is or may be COVID-19 infection

- ◯ Not relevant
- ◯ Slightly
- ◯ Moderately
- ◯ Very
- ◯ Extremely relevant

Note: if you have NOT answered/chosen at least one of the following items: [2] in question 3, skip the following question

Q217: Difficulty reaching clients and carers who are self-isolating or shielded currently

- ◯ Not relevant
- ◯ Slightly
- ◯ Moderately
- ◯ Very
- ◯ Extremely relevant

Note: if you have NOT answered/chosen at least one of the following items: [2] in question 3, skip the following question

Q218: Difficulties supporting clients who do not have their usual level of family support

- ◯ Not relevant
- ◯ Slightly
- ◯ Moderately
- ◯ Very
- ◯ Extremely relevant

Note: if you have NOT answered/chosen at least one of the following items: [2] in question 3, skip the following question

Q219: Difficulty engaging remotely with people with cognitive or sensory impairments

- ◯ Not relevant
- ◯ Slightly
- ◯ Moderately
- ◯ Very
- ◯ Extremely relevant

Note: if you have NOT answered/chosen at least one of the following items: [2] in question 3, skip the following question

Q220: Increased pressures because of reduced levels of social care, primary care, physical health and other community services supporting older people

- ◯ Not relevant
- ◯ Slightly
- ◯ Moderately
- ◯ Very
- ◯ Extremely relevant

Note: if you have NOT answered/chosen at least one of the following items: [2] in question 3, skip the following question

Q221: Increased need for involvement in end of life planning

- ○ Not relevant
- ○ Slightly
- ○ Moderately
- ○ Very
- ○ Extremely relevant

For staff working in or managing services for children and adolescents

How relevant has each of the following challenges been to you at work since mid-March 2020?

Rate from Not very relevant to Extremely relevant.

Note: if you have NOT answered/chosen at least one of the following items: [4] in question 3, skip the following question

Q222: Concerns resulting from withdrawal of the support and structure usually provided by schools

◯ Not relevant ◯ Slightly ◯ Moderately ◯ Very
◯ Extremely relevant

Note: if you have NOT answered/chosen at least one of the following items: [4] in question 3, skip the following question

Q223: Greater than usual safeguarding issues

◯ Not relevant ◯ Slightly ◯ Moderately ◯ Very
◯ Extremely relevant

Note: if you have NOT answered/chosen at least one of the following items: [4] in question 3, skip the following question

Q224: Greater difficulty in planning care because of reductions in other services, including health visiting and social care services

◯ Not relevant ◯ Slightly ◯ Moderately ◯ Very
◯ Extremely relevant

Note: if you have NOT answered/chosen at least one of the following items: [4] in question 3, skip the following question

Q225: Greater difficulty than usual in initiating and continuing child protection and children in need processes

◯ Not relevant ◯ Slightly ◯ Moderately ◯ Very
◯ Extremely relevant

Note: if you have NOT answered/chosen at least one of the following items: [4] in question 3, skip the following question

Q226: Challenges arising from increased family tension and conflicts

◯ Not relevant ◯ Slightly ◯ Moderately ◯ Very
◯ Extremely relevant

Note: if you have NOT answered/chosen at least one of the following items: [4] in question 3, skip the following question

Q227: Difficulty engaging children in remote appointments

- ◯ Not relevant
- ◯ Slightly
- ◯ Moderately
- ◯ Very
- ◯ Extremely relevant

For staff working in or managing services for people with drug and alcohol problems

How relevant has each of the following challenges been to you at work since mid-March 2020?

Rate from Not very relevant to Extremely relevant.

Note: if you have NOT answered/chosen at least one of the following items: [3] in question 3, skip the following question

Q228: Lack of access to usual detoxification support in residential settings

- ◯ Not relevant
- ◯ Slightly
- ◯ Moderately
- ◯ Agree
- ◯ Extremely relevant

Note: if you have NOT answered/chosen at least one of the following items: [3] in question 3, skip the following question

Q229: Difficulty in conducting community detoxifications due to infection control considerations

- ◯ Not relevant
- ◯ Slightly
- ◯ Moderately
- ◯ Agree
- ◯ Extremely relevant

Note: if you have NOT answered/chosen at least one of the following items: [3] in question 3, skip the following question

Q230: Increased demand due to sudden discharge from rehabilitation services

- ◯ Not relevant
- ◯ Slightly
- ◯ Moderately
- ◯ Agree
- ◯ Extremely relevant

Note: if you have NOT answered/chosen at least one of the following items: [3] in question 3, skip the following question

Q231: Disruption of methadone maintenance services

- ◯ Not relevant
- ◯ Slightly
- ◯ Moderately
- ◯ Agree
- ◯ Extremely relevant

Note: if you have NOT answered/chosen at least one of the following items: [3] in question 3, skip the following question

Q232: Greater risk of accidental overdose because large amounts of methadone have to be dispensed

- ◯ Not relevant
- ◯ Slightly
- ◯ Moderately
- ◯ Agree
- ◯ Extremely relevant

Note: if you have NOT answered/chosen at least one of the following items: [3] in question 3, skip the following question

Q233: Increased relapses under stresses of the current situation

- ◯ Not relevant
- ◯ Slightly
- ◯ Moderately
- ◯ Agree
- ◯ Extremely relevant

Note: if you have NOT answered/chosen at least one of the following items: [3] in question 3, skip the following question

Q234: Challenges in managing inadvertent withdrawal

- ◯ Not relevant
- ◯ Slightly
- ◯ Moderately
- ◯ Agree
- ◯ Extremely relevant

Note: if you have NOT answered/chosen at least one of the following items: [3] in question 3, skip the following question

Q235: Clients who have difficulty adhering to current guidance on social distancing, self-isolation or shielding

- ◯ Not relevant
- ◯ Slightly
- ◯ Moderately
- ◯ Agree
- ◯ Extremely relevant

For staff working in or managing perinatal services

How relevant has each of the following challenges been to you at work since mid-March 2020?

Rate from Not very relevant to Extremely relevant.

Note: if you have NOT answered/chosen at least one of the following items: [6] in question 3, skip the following question

Q236: Reduced access to maternity units to carry out assessments

- ◯ Not relevant
- ◯ Slightly
- ◯ Moderately
- ◯ Very
- ◯ Extremely relevant

Note: if you have NOT answered/chosen at least one of the following items: [6] in question 3, skip the following question

Q237: Difficulty planning and monitoring treatment due to reduced community midwife and health visitor services

- ◯ Not relevant
- ◯ Slightly
- ◯ Moderately
- ◯ Very
- ◯ Extremely relevant

Note: if you have NOT answered/chosen at least one of the following items: [6] in question 3, skip the following question

Q238: Difficulty planning and monitoring treatment due to reduced social care services

- ◯ Not relevant
- ◯ Slightly
- ◯ Moderately
- ◯ Very
- ◯ Extremely relevant

Note: if you have NOT answered/chosen at least one of the following items: [6] in question 3, skip the following question

Q239: Challenges assessing mother and infant relationships because of lack of direct access

- ◯ Not relevant
- ◯ Slightly
- ◯ Moderately
- ◯ Very
- ◯ Extremely relevant

Note: if you have NOT answered/chosen at least one of the following items: [6] in question 3, skip the following question

Q240: Referrals to our service not made or delayed because of the COVID-19 crisis

- ◯ Not relevant
- ◯ Slightly
- ◯ Moderately
- ◯ Very
- ◯ Extremely relevant

Note: if you have NOT answered/chosen at least one of the following items: [6] in question 3, skip the following question

Q241: Reduced opportunities to admit to mother and baby units

- ◯ Not relevant
- ◯ Slightly
- ◯ Moderately
- ◯ Very
- ◯ Extremely relevant

Note: if you have NOT answered/chosen at least one of the following items: [6] in question 3, skip the following question

Q242: Challenges arising from maternal or infant COVID-19 infection

- ◯ Not relevant
- ◯ Slightly
- ◯ Moderately
- ◯ Very
- ◯ Extremely relevant

Note: if you have NOT answered/chosen at least one of the following items: [6] in question 3, skip the following question

Q243: Safeguarding procedures are more difficult than usual to mobilise

- ◯ Not relevant
- ◯ Slightly
- ◯ Moderately
- ◯ Very
- ◯ Extremely relevant

Note: if you have NOT answered/chosen at least one of the following items: [6] in question 3, skip the following question

Q244: Children are too readily taken into care because of obstacles to making other assessment and management plans at the present time

- ◯ Not relevant
- ◯ Slightly
- ◯ Moderately
- ◯ Very
- ◯ Extremely relevant

For staff working in or managing forensic services

How relevant has each of the following challenges been to you at work since mid-March 2020?

Rate from Not very relevant to Extremely relevant.

Note: if you have NOT answered/chosen at least one of the following items: [5] in question 3, skip the following question

Q245: Greater seclusion because patients are not able to follow guidance

- ◯ Not relevant
- ◯ Slightly
- ◯ Moderately
- ◯ Very
- ◯ Extremely relevant

Note: if you have NOT answered/chosen at least one of the following items: [5] in question 3, skip the following question

Q246: Delayed discharges from forensic units because of restrictions in community leave or in access to community placements

- ◯ Not relevant
- ◯ Slightly
- ◯ Moderately
- ◯ Very
- ◯ Extremely relevant

Note: if you have NOT answered/chosen at least one of the following items: [5] in question 3, skip the following question

Q247: Greater obstacles to transferring prisoners into and out of the legal system

- ◯ Not relevant
- ◯ Slightly
- ◯ Moderately
- ◯ Very
- ◯ Extremely relevant

Note: if you have NOT answered/chosen at least one of the following items: [5] in question 3, skip the following question

Q248: Obstacles to assessing and treating patients because of reduced functioning of the legal system

- ◯ Not relevant
- ◯ Slightly
- ◯ Moderately
- ◯ Very
- ◯ Extremely relevant

Note: if you have NOT answered/chosen at least one of the following items: [5] in question 3, skip the following question

Q249: Patients/prisoners using COVID-19 as a weapon (e.g. by intentionally coughing on staff or other patients)

- ◯ Not relevant
- ◯ Slightly
- ◯ Moderately
- ◯ Very
- ◯ Extremely relevant

Note: if you have NOT answered/chosen at least one of the following items: [5] in question 3, skip the following question

Q250: Restricted access to prisons to manage mental health conditions among prisoners

○ Not relevant ○ Slightly ○ Moderately ○ Very
○ Extremely relevant

Note: if you have NOT answered/chosen at least one of the following items: [5] in question 3, skip the following question

Q251: Increased mental health morbidity and self-harm in prisons related to the current COVID-19 epidemic and its management

○ Not relevant ○ Slightly ○ Moderately ○ Very
○ Extremely relevant

Note: if you have NOT answered/chosen at least one of the following items: [5] in question 3, skip the following question

Q252: Has any provision been made for remote working with prisoners?

○ Yes ○ No

If yes, please tell us about this

For staff working in or managing services for people with intellectual disabilities and/or autism

How relevant has each of the following challenges been to you at work since mid-March 2020?

Rate from Not very relevant to Extremely relevant.

Note: if you have NOT answered/chosen at least one of the following items: [7] in question 3, skip the following question

Q253: Lack of high quality and relevant information for people with intellectual disabilities and/or autism about the COVID-19 pandemics and the requirements that result

- ◯ Not relevant
- ◯ Slightly
- ◯ Moderately
- ◯ Very
- ◯ Extremely relevant

Note: if you have NOT answered/chosen at least one of the following items: [7] in question 3, skip the following question

Q254: Difficulty for service users in comprehending the current crisis and the resulting requirements

- ◯ Not relevant
- ◯ Slightly
- ◯ Moderately
- ◯ Very
- ◯ Extremely relevant

Note: if you have NOT answered/chosen at least one of the following items: [7] in question 3, skip the following question

Q255: Increased need following the withdrawal of educational and support services in the community

- ◯ Not relevant
- ◯ Slightly
- ◯ Moderately
- ◯ Very
- ◯ Extremely relevant

Note: if you have NOT answered/chosen at least one of the following items: [7] in question 3, skip the following question

Q256: Difficulty engaging people with intellectual disabilities and/or autism with remote appointments

- ◯ Not relevant
- ◯ Slightly
- ◯ Moderately
- ◯ Very
- ◯ Extremely relevant

Note: if you have NOT answered/chosen at least one of the following items: [7] in question 3, skip the following question

Q257: Concerns about discrimination in access to physical health care for COVID-10

- ◯ Not relevant
- ◯ Slightly
- ◯ Moderately
- ◯ Very
- ◯ Extremely relevant

Note: if you have NOT answered/chosen at least one of the following items: [7] in question 3, skip the following question

Q258: Difficulty maintaining adequate levels of support for those with significant and complex needs

- ◯ Not relevant
- ◯ Slightly
- ◯ Moderately
- ◯ Very
- ◯ Extremely relevant

Note: if you have NOT answered/chosen at least one of the following items: [7] in question 3, skip the following question

Q259: Difficulty maintaining adequate support for families looking after a child/young person/adult with intellectual disabilities and/or autism who displays challenging behaviour

- ◯ Not relevant
- ◯ Slightly
- ◯ Moderately
- ◯ Very
- ◯ Extremely relevant

For staff working in or managing services for people with eating disorders

How relevant has each of the following challenges been to you at work since mid-March 2020?

Rate from Not very relevant to Extremely relevant.

Note: if you have NOT answered/chosen at least one of the following items: [8] in question 3, skip the following question

Q260: Difficulty monitoring weight remotely

- ◯ Strongly disagree
- ◯ Disagree
- ◯ Neither agree or disagree
- ◯ Agree
- ◯ Strongly agree

Note: if you have NOT answered/chosen at least one of the following items: [8] in question 3, skip the following question

Q261: Difficulty maintaining treatment programmes remotely

- ◯ Strongly disagree
- ◯ Disagree
- ◯ Neither agree or disagree
- ◯ Agree
- ◯ Strongly agree

Note: if you have NOT answered/chosen at least one of the following items: [8] in question 3, skip the following question

Q262: Exacerbation of eating disorders by current conditions during the COVID-19 epidemic, including changes in access to food and in meal routines

- ◯ Strongly disagree
- ◯ Disagree
- ◯ Neither agree or disagree
- ◯ Agree
- ◯ Strongly agree

Demographics

So that we can better understand the representation of our sample, please tell us your:

Q263: Gender

- ◯ Male
- ◯ Female
- ◯ Other
- ◯ Prefer not to say

Q264: Age

- ◯ Under 25
- ◯ 25 - 34
- ◯ 35 - 44
- ◯ 45 - 54
- ◯ 55 - 64
- ◯ 65 or over
- ◯ Prefer not to say

Q265: Ethnic group (please select from drop down menu below)

- ◯ BLANK
- ◯ White British
- ◯ White Irish
- ◯ White Gypsy or Irish Traveller
- ◯ White Any other background
- ◯ White and Black Caribbean
- ◯ White and Black African
- ◯ White and Asian
- ◯ Any other Mixed/Multiple ethnic background
- ◯ Indian
- ◯ Pakistani
- ◯ Bangladeshi
- ◯ Chinese
- ◯ Any other Asian ethnic background
- ◯ Black African
- ◯ Black Caribbean
- ◯ Any other Black/African/Caribbean background
- ◯ Arab
- ◯ Any other ethnic group
- ◯ Prefer not to say

Q266: Are you caring for any children under 18 at home?

- ◯ Yes
- ◯ No

Q267: Are you caring for any elderly or disabled relatives or friends?

- ◯ Yes
- ◯ No

Q268: Which best describes your current situation?

- ◯ Sick or self-isolating
- ◯ Working from home
- ◯ Mixture of workplace and working from home
- ◯ Mainly based at workplace

Q269: Do you think you have had COVID-19 infection?

- ◯ Yes, confirmed
- ◯ Yes, suspected
- ◯ No, not suspected

Q270: Do you think anyone in your household has had COVID-19 infection?

- ◯ Yes, confirmed
- ◯ Yes, suspected
- ◯ No, not suspected

Final page

Thank you so much for taking the time to participate.

Q271: Would you like to expand on any of the problems mentioned in this survey or tell us about any other significant problems that have arisen in your service during the COVID-19 period? If so, please describe here:

Q272: Would you like to tell us about any other innovation or adaptation that you or your team have made to cope with the situation? If so, please describe here:

Q273: Overall, in the area in which you work, are you aware of any major gaps in care provision that have not already been mentioned? If so, please describe here:

Q274: Have your experiences during the pandemic resulted in any significant learning for patient and staff safety?

◯ Yes ◯ No

If yes, please tell us about this if you don't mind

Q275: Is there any way in which the mental health care available at present in your area is better than usual?

◯ Yes ◯ No

If yes, please tell us about this

Q276: Would you be willing to complete another questionnaire to see how things are going in one to two months time?

◯ Yes ◯ No

If yes, please give us an email address on which you can be contacted:

Q277: We would also like to ask some people who have participated some further questions about particular aspects of the COVID-19 impact and response in which we are interested. This can be done by phone or video interview, or by email or Whatsapp, as suits you. Could we contact you about this? Like this questionnaire, it will be entirely confidential.

◯ Yes ◯ No

If yes, please give us your email address or tell us the best way to contact you:

